# Macrophage‐derived galectin‐3 contributes to pyroptosis, apoptosis and necroptosis through TLR4/MyD88/NF‐κB/NLRP3 during atherosclerosis

**DOI:** 10.1002/ctm2.70637

**Published:** 2026-03-08

**Authors:** Zihui Yuan, Haitao Li, Bing Xing Ruan, Hongyi Huang, Yiqing Li, Jian Wang

**Affiliations:** ^1^ Department of Vascular Surgery Union Hospital Tongji Medical College Huazhong University of Science and Technology Wuhan China; ^2^ University of Minnesota Twin Cities Twin Cities Minnesota USA

**Keywords:** apoptosis, atherosclerosis, galectin‐3, necroptosis, NLRP3, pyroptosis

## Abstract

**Background:**

Pyroptosis, apoptosis and necroptosis (PANoptosis) simultaneously occur and are extensively cross‐linked in infectious and inflammatory diseases. However, the co‐existence and regulation of macrophage pyroptosis, apoptosis and necroptosis in atherosclerosis have not yet been investigated.

**Methods:**

Atherosclerotic specimens from human lower extremity amputation and carotid endarterectomy were analysed. Ox‐LDL‐induced macrophages and high‐fat diet (HFD)‐fed *ApoE^−/−^* mice were employed as in vitro and in vivo models, respectively. Galectin‐3 was recognised as a key differentially expressed protein and gene related to PANoptosis by integrative proteomic and transcriptomic analysis of atherosclerotic murine aortas. Single‐cell transcriptomic analysis of human carotid endarterectomy specimens investigated the cellular distribution of galectin‐3. Galectin‐3 is a potent driver of macrophage activation and elicits inflammation through NLRP3 inflammasome activation. To elucidate the role of galectin‐3/NLRP3 in atherosclerosis, galectin‐3 siRNA transfection in macrophages was conducted, galec*tin‐3* and *ApoE* dual‐deficiency mice were produced, AAV‐F4/80‐shGalectin‐3 was injected, and NLRP3 agonist nigericin was administered.

**Results:**

A substantial content of inflammatory factors, the activation of NLRP3/GSDMD/CASP3/CASP8/RIPK3/pMLKL, and the upregulation of galectin‐3 were detected in advanced human and mouse atherosclerotic lesions. Galectin‐3 was predominantly expressed in atherosclerotic macrophages, and Galectin‐3‐positive macrophages were mainly distributed in the atherosclerotic core in comparison with the proximal adjacent artery. Ox‐LDL induced apoptosis, pyroptosis and necroptosis in macrophages, as evidenced by the activation of NLRP3/GSDMD/CASP3/CASP8/RIPK3/pMLKL and the secretion of proinflammatory cytokines. Galectin‐3 interacted with NLRP3. Genetic knockdown of galectin‐3 alleviated ox‐LDL‐induced activation of inflammatory cell death, which was pronouncedly abrogated by NLRP3 agonist nigericin. Genetic galectin‐3 deficiency attenuated, and conversely nigericin exacerbated macrophage death, vascular inflammation and atherosclerosis in HFD‐fed *ApoE^−/−^* mice. Mechanistically, galectin‐3 activated the TLR4/MyD88/NF‐κB/NLRP3 axis and induced pyroptosis, apoptosis and necroptosis in macrophages.

**Conclusions:**

Macrophage‐derived galectin‐3 contributed to pyroptosis, apoptosis and necroptosis in concert, promoted vascular inflammation and atherosclerosis through the upregulation of TLR4/MyD88/NF‐κB/NLRP3 pathway.

**Key points:**

- Pyroptosis, apoptosis, and necroptosis of macrophages occur concurrently in atherosclerosis.
- Galectin‐3 and NLRP3 expression levels are elevated in both human and murine atherosclerotic lesions.
- Galectin‐3 is predominantly expressed in macrophages within atherosclerotic plaques.
- Galectin‐3 interacts with NLRP3, activates TLR4/MyD88/NF‐_k_B/NLRP3 signal axis, and induces PANoptosis‐like cell death.
- Galectin‐3 deficiency attenuates, whereas the NLRP3 agonist nigericin exacerbates, atherosclerotic lesion development.

## Graphical abstract

Pyroptosis, apoptosis and necroptosis of macrophages occur concurrently during atherosclerosis. Galetein‐3 and NLRP3 are highly expressed in atherosclerotic lesions. Galectin‐3 is principally expressed in macrophages within atherosclerotic plaques. Galectin‐3 interacts with NLRP3, activates TLR4/MyD88/NF‐κB/NLRP3 signal axis, and promotes PANoptosis‐like cell death. Galectin‐3^−/−^ relieves, and NLRP3 agonist nigericin enlarges atherosclerotic lesions.

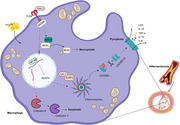

## 1 INTRODUCTION

Macrophages, the predominant cellular component of atherosclerotic lesions, play a crucial role in atherosclerotic plaque formation.^1^ Oxidised low‐density lipoprotein (Ox‐LDL) exerts cytotoxic effects and induces the death of macrophage foam cells in situ. Dead cells subsequently release proinflammatory intracellular contents to recruit and mobilise monocytes from bone marrow to the lesion sites, completing a vicious cycle that accelerates disease progression.^2^ Macrophage cell death accelerates necrotic core formation and increases plaque vulnerability.^2^

Necroptosis is a form of lytic cellular death that morphologically resembles cellular necrosis and is characterised by mitochondrial dysfunction, cellular swelling, plasma membrane rupture and local inflammatory process.^3^ Mechanically, receptor‐interacting protein kinase‐1 (RIPK1) cooperates with RIPK3 to activate it, which subsequently recruits and phosphorylates the pseudokinase mixed lineage kinase domain‐like (MLKL).^4^ The pMLKL forms pores in the membrane and functions as a pore‐forming complex, directly inducing necrotic membrane disruption.^5^ Pyroptosis, an inflammatory cellular death, is accompanied by cellular oedema, plasma membrane pore formation, causing the release of proinflammatory cytoplasmic molecules containing interleukin (IL)‐1β and IL‐18.^6^ Mechanically, canonical caspase‐1 activation upon the nod‐like receptor family pyrin domain containing 3 (NLRP3) inflammasome cleaves gasdermin D (GSDMD) into its active form, amino‐terminal GSDMD.^7^ The N‐terminal GSDMD‐N domain, a pore‐drilling fragment, results in the perforation on membrane and ultimately causes pyroptosis.^8^ Various cytotoxic death stimuli linked to atherosclerotic lesions can lead to cellular pyroptosis and necroptosis.^9^ Apoptosis is the process of regulated cellular death and is initiated by the activation cascade of caspases. Macrophage apoptosis reduces cellularity and progression in early atherosclerotic lesions, but even promotes plaque necrosis in advanced lesions.^10^, ^11^ Necroptosis, pyroptosis and apoptosis occur concurrently and show the extensive crosstalk among three death pathways during infectious diseases, autoimmune conditions and malignant tumours,^12^ resulting in the concept of PANoptosis. The PANoptosome complex, which triggers this process, is assembled by key modulators shared across necroptosis, pyroptosis and apoptosis. The co‐existence of necroptosis, pyroptosis and apoptosis has been documented in retinal neuronal ischaemia/reperfusion injury^13^ and liver functional failure.^14^ Atherosclerosis is implicated in pyroptosis, apoptosis and necroptosis in macrophages. Here, we first test whether pyroptosis, apoptosis and necroptosis are cooperatively and simultaneously activated in advanced atherosclerosis.

We incorporated the GSE83112 dataset from Gene Expression Omnibus (GEO), and performed a bulk transcriptomic bioinformatics analysis to identify differentially expressed genes (DEGs) between atherosclerotic and normal murine aortas. Proteomic analysis identified differentially expressed proteins (DEPs) in the atherosclerotic aorta of high‐fat diet (HFD)‐fed *ApoE^−/−^* mice compared to *ApoE^−/−^* mice. Comparison of DEGs and DEPs revealed three shared molecules with increased abundance in the murine atherosclerotic aortas compared to normal aortas. We downloaded the mRNA‐sequencing dataset GSE111782 of human carotid artery endarterectomy plaques. Three shared transcriptomes and proteomes, including Lgals3/Galectin‐3, Ctss/Cathepsin‐S and Vcam1, were strongly upregulated in human atherosclerotic plaques exhibiting elevated expression of PANoptosis markers. We utilised single‐cell transcriptomic data from GSE159677 and revealed that galectin‐3‐positive macrophages were more extensively distributed within the atherosclerotic core of human carotid plaques.

Galectin‐3, encoded by Lagls3 in the genome, participates in multiple biological activities, including cellular adhesion, activation, proliferation, migration and apoptosis.^14^ Serum galectin‐3 levels may function as a valuable bioindicator for unstable coronary artery disease and cardiac modality.^15^, ^16^ There are several conflicting reports concerning the role of galectin‐3 in atherosclerosis. Genetic deletion and pharmacological inhibition of galectin‐3 have been demonstrated to attenuate the development of atherosclerotic plaques.^17^ Conversely, galectin‐3 exerts a significant protective effect on atherosclerosis by depressing macrophage polarisation and their invasive capacity.^18^ The NLRP3 inflammasome is activated in macrophages by ox‐LDL, thereby contributing to atherogenesis in the vessel wall.^19^ The NLRP3 inflammasome is critical for the congregation of the PANoptosome complex and the subsequent initiation of pyroptosis, apoptosis and necroptosis.^20^ Galectin‐3 interacts with NLRP3 and regulates the proinflammatory cascades in macrophages.^21^, ^22^ The second aim is to test whether galectin‐3/NLRP3 can be therapeutically targeted for the depression of atherosclerosis by regulating macrophage necroptosis, pyroptosis and apoptosis in preclinical animal models of atherogenesis. Next, we identified macrophage subpopulations exhibiting unique gene expression profiles using a single‐cell RNA sequencing (scRNA‐seq) dataset obtained from human carotid endarterectomy specimens. GO‐biological process (BP) and KEGG pathway analysis of DEGs in macrophages revealed that the programmed cell death in macrophage subpopulations might be linked to myeloid differentiation primary response protein 88 (MyD88)‐dependent Toll‐like receptor 4 (TLR4) and nuclear factor kappa (NF‐κB) signalling pathways. The third aim is to determine whether the TLR4/MyD88/NF‐κB axis participates in pyroptosis, apoptosis and necroptosis in macrophages under the co‐regulation of galectin‐3/NLRP3.

## 2 MATERIALS AND METHODS

### 2.1 Proteomics analysis

The samples were homogenised with SDT buffer, and then the lysate was homogenised using a high‐speed benchtop homogeniser. The homogenate was subjected to sonication, followed by centrifugation at 13 000 × *g* for 20 min, and the supernatant was subjected to filtration through.22 µm membrane filters. The protein concentration of the filtrate was measured using the bicinchoninic acid (BCA) assay. Twenty micrograms of protein from the indicated sample were mixed with 6× loading buffer. The proteins were then resolved in a 10%–12% SDS‐PAGE gel. Protein bands were visualised with Coomassie brilliant blue staining. Aliquots of 100 µg of proteins were deoxidised with 20 mM DTT. UA buffer was used to remove the detergent, DTT and other low‐molecular‐weight components via repeated ultrafiltration. Then 100 µL iodoacetamide was added for cysteine alkylation, and the samples were treated in the dark. Finally, protein suspensions were digested with trypsin, ultrafiltered, and the soluble peptides were then obtained. The peptide concentrations were calculated by measuring the absorption value at 280 nm UV light.

LC‐MS/MS analyses were carried out on a high‐resolution Q‐Orbitrap Exploris 480 mass spectrometer. Approximately 500 ng of peptides were directly applied onto a trap column and separated by chromatography. All MS data were obtained in a data‐dependent acquisition mode using a top 10 method by dynamically choosing the most abundant precursor ions from the survey scan (300–1800 m/z). MS1 survey scans were performed with a resolution of 120 000, a normalised automatic gain control (AGC) target value of 300% and a maximum injection time (IT) of 50 ms. MS2 scans were carried out at 15 000 resolution and with AGC target value at 75%, and maximum IT of 35 ms, and isolation width was set to 1.6 m/z.

The extracted MS data were processed using MaxQuant software version 1.6.17.0. For protein identification, the raw MS data were processed by searching against the protein database. The search was performed according to the cleavage specificity of trypsin. Up to two missed cleavage sites of trypsin were allowed. The search mass tolerance was set as 20 ppm for fragment ions. Carbamidomethylation of cysteine residues was specified as a fixed modification, while acetylation on protein N‐terminal and oxidation on methionine were specified as variable modifications. False discovery rate (FDR) of peptide was established at.01. The relative expression abundance of proteins was determined based on the normalised spectral abundance factor. Proteins with *p*‐value less than.05 and fold‐change greater than 2 or less than.5 were considered as significantly differentially expressed proteins.

### 2.2 Bioinformatic analysis

The atherosclerosis‐related gene expression profiles of the GSE83112 dataset were obtained from the Gene Expression Omnibus (GEO) database. This dataset consisted of three aortic tissues obtained from *ApoE^−/−^* mice fed a normal diet (ND) (GSM2193157, GSM2193158 and GSM2193159) and three atherosclerotic aortas collected from *ApoE^−/−^* mice fed a high‐fat diet (HFD) for 8 weeks (GSM2193160, GSM2193161 and GSM2193162). The atherosclerosis group was carefully matched with the normal control group to discover differentially expressed genes (DEGs).

Analyses were carried out in R version 4.0.3. Gene probe IDs in expression profiles were remodelled into the gene's symbol using the platform‐specific annotation files. Probe set identifiers corresponded to the gene's symbol. After several probes were indexed to the unique gene identifier, the expression levels of those probe sets were averaged. When one probe was matched to multiple genes, it was deleted. DEGs were determined using the limma package with the criteria of *p*‐value less than.05 and Log2 FoldChange ≥1. Gene Ontology (GO) enrichment is ubiquitously used for interpreting the functional characteristics of a set of genes by making use of the GO system of classification (http://geneontology.org/). GO enrichment analysis of DEGs was performed with the cluster Profiler R package, and length bias in gene expression was amended during this process. GO terms exhibiting a corrected *p*‐value below.05 were proven to be markedly enriched. Kyoto Encyclopedia of Genes and Genomes (KEGG) linked genomes and potential signalling pathways. Similarly, the cluster Profiler R package was utilised to test statistically enriched DEGs in KEGG pathways.

### 2.3 Consensus clustering

The GSE111782 dataset, available through the GEO platform, offered the gene expression profiles of human carotid artery atherosclerotic plaques. This dataset included nine symptomatic carotid plaques (GSM3039648–GSM3039656) and nine asymptomatic carotid plaques (GSM3039657–GSM3039665). The downloaded raw probe expression matrix was then remodelled into a gene expression matrix. Raw array data were normalised with each group using robust multichip average (RMA). Multiple probes corresponding to one gene were averaged as the gene expression value.

By means of literature search, we identified genes that were significantly linked to pyroptosis, apoptosis and necroptosis (PANoptoosis), including ZBP1, RIPK1, RIPK3, FADD, CASP8, NLRP3, ASC, CASP1, GSDMD, MLKL and NINJ1. According to the expression of PANoptosis‐related genes, a resampling‐based validity scheme known as consensus clustering was employed for cluster identification in the GSE111782 dataset. Cluster analysis was carried out using the ‘ConsensusCluster Plus’ R package. Subsequently, the consensus cumulative distribution function (CDF) curve, CDF delta area curve and consensus clustering matrix were jointly utilised to generate different *k* values for determining the optimal number of clusters. We generated two clusters based on the genes related to PANoptosis. Additionally, the ‘Limma’ package R program was applied to determine the DEGs between two clusters, and adjusted *p*‐value below.05 and Log2 FoldChange ≥1 were explained as the threshold. We performed GO and KEGG enrichment analyses on the DEGs between the two clusters using the ‘ClusterProfiler’ R package. Based on the normalised gene expression data, immune cell infiltration in clusters was evaluated by ESTIMATE and CIBERSORT to explore the disease immune microenvironment. Immune cell infiltration of 22 distinct immune cells was available in the databases encompassing 547 immune‐specific markers across two clusters. The pattern of 22 kinds of immune cell infiltration was clarified using CIBERSORT. The number of random sample permutations was set at 1000. Then the ESTIMAE score, immune and stromal score were calculated by the ESTIMATE algorithm.

### 2.4 Cell culture and treatment

THP‐1 monocytes were sourced from Procell Life Science & Technology Co. Ltd (Wuhan, China). Cells were maintained in DMEM or complete PRMI‐1640 medium supplemented with 10% fetal bovine serum (FBS) in a humidified atmosphere of 95% air and 5% CO_2_ at 37°C. THP‐1 cells were incubated in six‐well cell culture plates at a density of 1 × 10^6^ cells per well for 48 h treatment with 100 nm PMA to activate the differentiation of monocytes into macrophages. THP‐1 macrophages were exposed to 100 µg/mL ox‐LDL for 24–48 h for the in vitro formation of foam cells. For pharmacological management, THP‐1 macrophages were treated with ox‐LDL and were stimulated by NLRP3 agonist nigericin (10 µm) for 1 h or berubicin (1 µm) for 8 h before being harvested.

### 2.5 RNA interference

The siGalectin‐3 (NCBI Gene ID: 3958) and siControl were obtained from RiboBio Co. Ltd (Guangzhou, China). Three pairs of RNA interference sequences targeting Lgals3/galectin‐3 gene and corresponding control sequences were designed and chemically synthesised. Target sequences for the siGalectin‐3 were as follows: siGalectin‐3_1, CCTTAGCTGGCTCTGGAAA; siGalectin‐3_2, GCATGCTGATCACAATCAT; and siGalectin‐3_3, GGTTGCGGTCAATGATGTT. Before ox‐LDL treatment, THP‐1 macrophages were incubated in vitro and infected with siControl or siGalectin‐3 using transfection reagent. All cells were inoculated into a 96‐well plate for 24 h following transfection of siRNA. Knockdown efficiency of galectin‐3 in macrophages was measured by RT‐qPCR. The mRNA levels of galectin‐3 were markedly decreased in macrophages transfected with siGalectin‐3_1 and siGalectin‐3_3 compared to those transfected with siControl. The siGalectin‐3_1 against CCTTAGCTGGCTCTGGAAA most productively suppressed target gene and was applied for the subsequent experiments.

### 2.6 Plasmid construction

Vectors constructed on the adeno‐associated virus (AAV) encoding shRNA targeting the mouse Lgals3/galectgin‐3 gene sequence (NCBI Gene ID: 16854; GeneBank ID: NM_001145953.1) (5′‐CAACGATGCTCACCTACTGCA‐3′), the mouse TLR4 gene sequence (NCBI Gene ID: 21898; GeneBank ID: NM_021297.3) (5′‐CACTTTGTTTGCTCCTGCGAA‐3′), and negative control (5′‐TTCTCCGAACGTGTCACGT‐3′) were successfully synthesised and subsequently cloned into GV407 (pAAV‐F4/80p‐EGFP‐MIR155(RNAi)‐SV40) vector linearised with BsmBI restriction sites (Shanghai Genechem Co. Ltd.). The insertion of the gene in the recombinant vector was confirmed through restriction enzyme analysis and DNA sequencing.

### 2.7 Adeno‐associated virus production

The pHelper and pRC plasmids were transferred together into 293T cells using the Lipofectamine 3000 together with viral vectors. After 72 h of transfection, AAVs were harvested, and AAV9 purification was completed using an iodixanol column gradient ultracentrifuge, and the quantification of AAV concentration was subsequently achieved through a titre. Purified recombinant AAV viruses were typically propagated in AD293 cells and titrated with a quantitative PCR‐based approach. The AAV9 used in this study was stored in.001% Pluronic F‐68 solution.

### 2.8 In vitro AAV transduction

RAW 264.7 mouse mononuclear macrophages and mouse aortic endothelial cells (ECs) were sourced from Procell Life Science & Technology Co. Ltd (Wuhan, China). Mouse aortic smooth muscle cells (SMCs) were purchased from Warner Biotechnology Co. Ltd (Wuhan, China). Cells were seeded into 24‐well plates 24 h prior to infection. When cells reached approximately 30%–40% of confluence, they were transfected with 300 µL of AAV viral suspension. The values of multiplicity of infection (MOI) were set at 1 × 10^5^,.5 × 10^5^ and.3 × 10^5^ for macrophages, SMCs and ECs, respectively. After the cells were incubated at 37°C for 48 h, the supernatant was discarded and replaced with fresh medium. RNA samples were harvested on Day 5 post‐infection, and protein lysates were collected on Day 7 post‐infection to evaluate infection efficiency by qRT‐PCR and Western blotting. After RAW 264.7 macrophages were transfected with AAV‐F4/80‐shTLR4 or AAV‐empty vector and infection efficiency was confirmed, the cells were exposed to 100 µg/mL ox‐LDL to induce foam cell formation.

### 2.9 Caspase‐3 activity assay

Cell pellets were washed and resuspended in pre‐cooled cell lysis buffer, followed by incubation on ice for 30 min. To ensure thorough homogenisation, the tissue was first minced into small fragments using sterile scissors. Afterwards, the minced tissue was homogenised using a high‐throughput tissue crusher and further incubated with pre‐cooled cell lysis buffer for 30 min. Both cell lysate and tissue homogenate were centrifuged at 1100 × *g* for 10–15 min at 4°C. The resulting supernatant was subsequently collected for caspase‐3 activity measurement using a Colorimetric Assay Kit (E‐CK‐A383, Elabscience Biotechnology Co. Ltd, Wuhan, China).

### 2.10 Animal experiments

Apolipoprotein E‐deficient mice (*ApoE^−/−^*) with a C57BL/6J genetic background (4‐week old) were sourced from Changzhou Cavens Experimental Animal Co. Ltd (Changzhou, China). *Galectin 3^−/−^/ApoE^−/−^* mice with a C57BL/6J genetic background were purchased from Cyagen Biosciences Inc. (Suzhou, China). Cryopreserved sperm from Lgals/galectin‐3 heterozygous knockout mice were thawed and utilised for in vitro fertilisation with oocytes obtained from homozygous *ApoE^−/−^* female mice. The fertilised embryos obtained were subsequently transferred into pseudopregnant surrogate dams to produce *Galectin‐3^+/−^/ApoE^+/−^* mice. These mice were subsequently crossed with *ApoE^−/−^* mice to generate *Galectin‐3^+/−^/ApoE^−/−^* mice. Finally, galec*tin‐3^+/−^/ApoE^−/−^* mice were intercrossed, and the progeny were screened to identify *Galectin 3^−/−^/ApoE^−/−^* mice. Mice were raised in a room with relative humidity (40%–70%), controlled temperature (21°C–25°C) and an artificial light‐dark (12–12 h) cycle. The water and food were freely available.

For developing atherosclerosis, 6‐week‐old male *ApoE^−/−^* mice were fed an atherogenic high‐fat diet (HFD, catalogue #D12108C, Research Diets Inc., New Brunswick, NJ) for 4 months, consisting of 79.7% of basal diet, 20% of fat and.3% of cholesterol. A total number of 32 *ApoE^−/−^* mice and 8 *Galectin 3^−/−^/ApoE^−/−^* mice participated in our research. *ApoE^−/−^* mice (*n* = 8) in the control group were fed with a normal diet and administered intraperitoneally with homologous saline three times a week. Twenty‐four *ApoE^−/−^* mice were randomly assigned to the model group (*n* = 16) and NLRP3 agonist group (*n* = 8). For the modelling of atherosclerosis, *ApoE^−/−^* mice (*n* = 8) were maintained on an HFD for 4 months. For the study of NLRP3 agonist, *ApoE^−/−^* mice (*n* = 8) received 250 µL volume of a 5 mg/kilogram bodyweight dose of nigericin (dissolved in saline as vehicle) intraperitoneally three times a week and simultaneously with the HFD for 4 months.^23^ *Galectin 3^−/−^/ApoE^−/−^* mice (*n* = 8) were fed on an HFD for 4 months.

The AAV‐based shRNA knockdown system is an efficient viral vector for in vivo gene silencing. Macrophage‐specific galectin‐3 or TLR4 knockdown was achieved using recombinant AAV serotype 9 (AAV9). *ApoE^−/−^* mice (*n* = 24) were randomly assigned to receive tail‐vein microinjection at 4 weeks of age with one of the following AAV9 vectors: (i) the AAV9‐scramble control short hairpin RNA (shRNA) (AAV‐empty vector); (ii) macrophage‐specific AAV9‐galectin‐3 shRNA (AAV‐F4/80‐shGalectin‐3); (iii) macrophage‐specific AAV9‐TLR4 shRNA (AAV‐F4/80‐shGalectin‐3) (GeneChem, Shanghai, China) via tail vein at the age of 4 weeks. The shRNA sequences targeting galectin‐3 and TLR4 were 5′‐CAACGATGCTCACCTACTGCA‐3′ and 5′‐CACTTTGTTTGCTCCTGCGAA‐3′, respectively. Two weeks later, the above mice were placed on an HFD for 4 months.

On completion of these experiments, the mice were euthanased with sodium pentobarbital anaesthesia. Aortas were fixed en face and then stained with Oil Red O. The hearts were removed, frozen and sectioned into aortic root for Oil Red O, HE (haematoxylin and eosin) and Movat's staining.

### 2.11 Clinical tissue specimens

Carotid atheromatous plaque samples were obtained from three patients who underwent a carotid endarterectomy procedure. Advanced atherosclerotic lesions in the femoral arteries of the lower extremities were obtained from three individuals, each of whom encountered the necrotic amputation of limbs secondary to a major arterial occlusion. Three patients suffered from lower limb amputation due to malignant bone tumour, and the femoral artery was regarded as a histologically normal artery devoid of atherosclerosis. Clinical specimens were immediately immersed in liquid nitrogen for preservation or alternatively stored at −80°C for further analysis. All patients provided explicit written informed consent before surgical procedures.

### 2.12 Histological examination

Mice hearts and proximal aortic root were excised and vertically embedded in optimal cutting temperature (OCT) compound. The aortic sinus was serially cryosectioned in 5 µm slices, and the sections corresponding to the same anatomical slices of each heart were operated on. Human histological samples were rapidly frozen in liquid nitrogen, embedded in OCT media, and sectioned into 5 µm pieces. Histological staining involved HE and Movat's pentachrome stainings.

### 2.13 Oil Red O staining

Cells were fixed in 4% paraformaldehyde, rinsed with 60% isopropanol, and stained with.5% Oil Red O solution. The stained cells were then washed with sterile water to remove any excess dye, observed under the microscope with conventional light and photographed.

Frozen sections at 5 µm thickness were prepared from human specimens and mouse hearts in a cryostat and fixed with 4% paraformaldehyde in PBS for 15 min. The sections were then stained with pre‐warmed Oil Red O solution, differentiated with 60% isopropanol, and further counterstained with haematoxylin. The slides were examined using a light microscope to enable clear visualisation and quantification of lipid‐rich regions.

The whole aorta was fixed by immersing it in 4% paraformaldehyde. After the elimination of the surrounding appendage, the entire aorta from its arch to the bifurcation into the two common iliac arteries was cut open along the longitudinal direction, processed with.5% Oil Red O working solution, and subsequently rinsed with 60% isopropanol.

### 2.14 Transmission electron microscopy (TEM)

After cells were stimulated and tissues were freshly collected, the samples were preserved in 2.5% glutaraldehyde‐paraformaldehyde to stabilise ultrastructure. Cells were scraped off the plates and collected into centrifuge tubes. Cell blocks and tissues were then fixed with 1% osmic acid, dehydrated with ascending ethanol concentrations (50%, 60%, 70%, 80%, 90%, 95% and 100%), and penetrated with propylene oxide. The sections were prepared using an ultrathin microtome and stained en bloc with uranyl acetate and lead citrate.

### 2.15 Co‐immunoprecipitation (Co‐IP)

After the stimulation of ox‐LDL, the pelleted cells were resuspended in lysis buffer with phosphatase inhibitors and protease inhibitors. The whole cell lysates were sonicated and centrifuged at 12 000 × *g* for 15 min. Then the supernatant was incubated overnight with two antibody combinations: (i) anti‐NLRP3 (30109‐1‐AP, Proteintech) and anti‐galectin 3 (60207‐1‐Ig, Proteintech); (ii) anti‐TLR4 (19811‐1‐AP, Proteintech) and anti‐MyD88 (67969‐1‐Ig, Proteintech). Purified immunoglobulin G (IgG) was employed as the negative control for IP. Subsequently, protein A/G conjugated with magnetic agarose beads was combined with the mixture, and it was incubated and shaken continuously. The solutions were then separated from the beads using the magnetic separation rack. Finally, the IP complexes were eluted with elution buffer and subsequently subjected to immunoblotting (IB).

### 2.16 Annexin V‐FITC/propidium iodide (PI) apoptotic assay

Cell apoptosis was validated by flow cytometry using an annexin V‐FITC/PI apoptosis detection kit. Briefly, cells were subjected to diverse treatments. The cells were then digested by trypsinisation and labelled with annexin V‐FITC/PI. Annexin V and PI double staining was conducted to examine the mode of cellular death. Early‐phase apoptosis was solely positive for annexin V staining, while late‐phase apoptosis and pyroptosis were marked by both FITC annexin V and PI positive stainings.

### 2.17 Flow cytometry analysis of PI staining

After treatment, the cells were digested with trypsin and centrifuged for 5 min. These cells were resuspended and stained with a mixed working solution of Hoechst 33342 for live cells and 2 µg/mL PI for 20 min at 37°C to mark cells with membrane pores. Pyroptotic macrophages were detected by flow cytometry, with the proportion of pyroptotic cells expressed as the ratio of the number of stained cells to the total number of cells.

### 2.18 Lactate dehydrogenase (LDH) test

Membrane integrity was evaluated by measuring LDH leakage from cells. LDH activity in the cell supernatants was measured using a commercial detection kit (Jiancheng, Nanjing, China). Briefly, cells were grown in 12‐well plates and treated with drugs and/or siRNAs. After the cell culture medium was removed, the working solution was added to each well and incubated for 30 min. Then, the reaction was terminated by the addition of the stop solution to each well. The absorbance value was measured at a wavelength of 450 nm.

### 2.19 Western blotting analysis

Tissues or cells were lysed on ice in RIPA lysis buffer. The lysates were then sonicated and centrifuged, and the cleared supernatants were collected. The final protein concentrations in the supernatant were measured using the BCA method. Aliquots of protein per sample were run on SDS‐PAGE gels and transferred to PVDF membranes.

Membranes were then incubated overnight on a shaker with primary mouse or rabbit antibodies against galectin‐3 (60207‐1‐Ig, Proteintech, China), GSDMD (AF4012, Affinity Biosciences, China), NLRP3 (DF7438, Affinity Biosciences, China), caspase‐3 (66470‐2‐lg, Proteintech, China), caspase‐8 (66093‐1‐Ig, Proteintech, China), RIPK3 (A5431, ABclonal, China), MLKL (A26436, ABclonal, China), Phospho‐MLKL (AP0949, ABclonal, China), TLR4 (GB11519, Servicebio, China), MyD88 (GB12269, Servicebio, China), NF‐κB (10745‐1‐AP, Proteintech, China), Phospho‐NF‐κB (GB113882, Servicebio, China) and GAPDH (60004‐1‐Ig, Proteintech, China). After washing, the membranes were sequentially blotted with horseradish peroxidase (HRP)‐conjugated anti‐mouse or rabbit secondary antibodies. Immunoreactive bands were detected using chemiluminescent detection reagents. Band intensities normalised to GAPDH were used to determine the relative protein expression.

### 2.20 Immunofluorescent staining

Human histological samples and mouse aortas were fixed with 4% paraformaldehyde, dehydrated, embedded in paraffin and sectioned at a thickness of 5 µm. Next, the sections were deparaffinised, and endogenous peroxidase activity was eliminated with 3% hydrogen peroxide, followed by blocking with 5% bovine serum albumin (BSA). Colocalisation of different antigens was performed using double or triple immunohistochemistry staining. Slides were incubated with combinations of different primary antibodies against GSDMD (AF4012, Affinity Biosciences, China), caspase‐3 (66470‐2‐lg, Proteintech, China), RIPK3 (A5431, ABclonal, China), F4/80 (GB113373, Servicebio, Wuhan, China) and CD68 (GB11063‐2, Servicebio, Wuhan, China). Slides were incubated with Alexa Fluor 594, Alexa Fluor 488 and Alexa Fluor 555 conjugated second antibodies (IgG).

The cells were grown in chamber slides, fixed in 4% formaldehyde, permeabilised with.1% Triton X‐100 and blocked with 1% BSA. The cells were then co‐immunostained with the following pairs of primary antibodies: (i) anti‐caspase‐3 (66470‐2‐lg, Proteintech, China) and anti‐RIPK3 (A5431, ABclonal, China); (ii) anti‐caspase‐3 (66470‐2‐lg, Proteintech, China) and anti‐GSDMD (AF4012, Affinity Biosciences, China). Next, the cells were immunostained with an Alexa Fluor 594 and Alexa Fluor 488‐conjugated IgG secondary antibodies. To investigate the NF‐κB nuclear translocation, the cells were incubated with an anti‐NF‐κB primary antibody (10745‐1‐AP, Proteintech, China), followed by Alexa Fluor 488‐conjugated IgG secondary antibodies. Confocal microscopy was used to acquire fluorescence images.

### 2.21 Cytokine assay by ELISA

Intermediate blood samples were collected from the retro‐orbital capillary sinus of mice. The serum was prepared by centrifugation of the whole blood at 3000 rpm for 15 min. TNF‐α, IL‐6, IL‐1β and IL‐18 in the supernatant were detected by ELISA assay.

### 2.22 Single‐cell RNA sequencing data handling

The scRNA‐seq dataset was downloaded from GSE159677 in the GEO database. Proximal adjacent (PA) carotid artery tissues and atherosclerotic core (AC) plaques were collected from three patients after carotid endarterectomy and were sequenced on an Illumina Nextseq 500 sequencing system using the Chromium instrument (10× Genomics read depth). Gene expression patterns of different cell subtypes were exported and processed using the Seurat software package (Version 4.2.0) in the R environment. The low‐quality cells were removed and did not participate in the downstream data analysis, especially those with fewer than 500 or more than 5000 genes per cell, and those with more than 20% mitochondrial gene expression. Gene expression counts were normalised using the *NormalizeData* function, the top 2000 highly variable genes were selected using the *FindVariableFeatures* function, and data scaling was carried out using the *ScaleData* function. Datasets were merged, and batch effects across datasets were corrected by applying *FindIntegrationAnchors* and *IntegrateData* functions. Principal component analysis (PCA) was conducted on 2000 genes using the *RunPCA* function, and principal components (PCs) were identified on highly variable genes. A shared nearest neighbour (SNN)‐based graph was established based on the top 30 PCs using *Findneighbors* function and was used to identify cell clusters using *Findclusters* function at a resolution of.6. Dimensionality reduction clustering was visualised using the *RuntSNE* and *RunUMAP* functions. The *FindAllMarkers* function was utilised to select marker genes specific for each cluster. Cell clusters were annotated manually based on canonical known marker genes. Differentially expressed genes (DEGs) were selected using the *FindMarkers* function with an absolute log‐fold threshold of.25 (log2FoldChange >.25) and a parameter min.pct of.25 from the default.1 to ensure that the detected genes are present in at least 25% of cells of the target clusters (pct.1 >25%). Genes with *p* < .05 in the differential genes list were subsequently used for GO function and KEGG enrichment analysis by *clusterProfiler* software. For gene set enrichment analysis (GSEA), all DEGs were ranked according to an absolute log2FC in descending order. GSEA analysis was conducted according to the GO database by using the gsea function implemented in the *clusterProfiler* package to identify which gene term was indeed significantly enriched in each individual cell cluster.

### 2.23 Statistical analysis

All statistical analyses were computed using GraphPad Prism 9.5 Software. Data were expressed as mean ± standard deviation (SD) from at least three independent repetitions. An unpaired, two‐tailed Student's *t*‐test was performed when comparing the means of two independent groups. One‐way analysis of variance (ANOVA) was conducted for comparison of multiple conditions. Statistical significance thresholds were indicated as ^*^ *p *˂ .05, ^**^ *p* ˂ .01, ^***^ *p* ˂ .001, ns = no significance.

## 3 RESULTS

### 3.1 DEPs are identified by proteomic analysis of mouse atherosclerotic aortas

**FIGURE 1:**
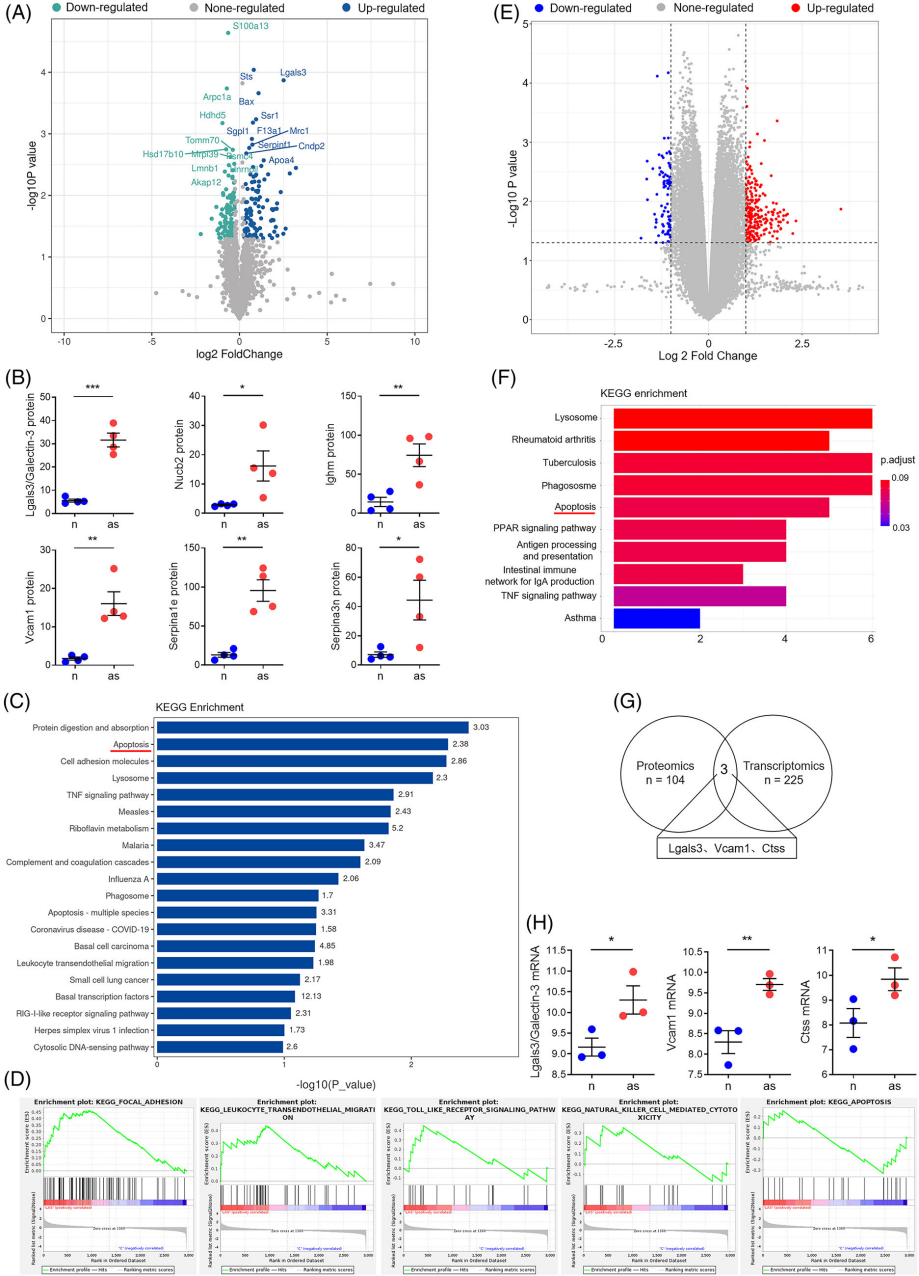
Identification of DEPs and DEGs by proteomic and transcriptome analysis of the aortas of *ApoE^−/−^* mice versus HFD‐fed *ApoE^−/−^* mice. (A) Volcano plot analysis represents the up‐ or downregulated DEPs abundance changes in the aortas from *ApoE^−/−^* mice versus HFD‐fed *ApoE^−/−^* mice. Blue indicates upregulated genes, green indicates downregulated genes, and grey indicates genes with unchanged genes. (B) The top six upregulated DEPs consisting of lgals3/galectin‐3, nucb2, ighm, vcam1, serpina1e and serpina3n between *ApoE^−/−^* mice and HFD‐fed *ApoE^−/−^* mice. (C) Numbers of DEGs are enriched in the identified pathway. Apoptosis is identified as the critical cell death forms. The *x*‐axis represents the number of enriched genes, and the *y*‐axis represents the name of the enriched KEGG pathway. (D) Compared to *ApoE^−/−^* mice, the gene set upregulated in HFD‐fed *ApoE^−/−^* mice is enriched in the signal pathways including foal adhesion, leukocyte transendothelial migration, Toll‐like receptor signalling pathway, natural killer cell mediated cytotoxicity, and apoptosis. (E) The Volcano plot illustrates that gene transcripts with a log2 fold change greater than 1 and a significant *p*‐value less than.05 are differently expressed between normal and atherosclerosis. Red represents upregulation, blue represents downregulation, and grey represents no change. (F) KEGG enrichment of DEGs indicates that apoptosis emerged as the fifth pathway of significant alteration. KEGG pathway analysis is performed with bar plot. Colours correspond to the *p*‐value, with red indicates more significant enrichment. (G) Venn diagram is performed to screen out the overlapping DEPs and DEGs. (H) The coordinated DEPs and DEGs, consisting of Galectin/Lgals3, Vcam1 and Ctss, are differentially expressed between *ApoE^−/−^* control mice and HFD‐fed *ApoE^−/−^* mice.

To investigate how HFD changes the proteome of the aorta of *ApoE^−/−^* mice and to comprehensively explain the underlying molecular mechanisms driving atherogenesis, Pearson correlation test of protein expression values across samples was performed to evaluate the consistency and the reproducibility among the four biological replicates, and the data were indicative of considerable variations in the protein levels of the aorta in *ApoE^−/−^* control mice versus HFD‐fed *ApoE^−/−^* mice (Figure S1A). Principal component analysis (PCA) demonstrated clear separation and minimal overlap between normal and atherosclerosis groups, indicating that the classification model was reliable (Figure S1B). Heatmaps demonstrated that *ApoE^−/−^* mice fed with ND and HFD exhibited unique protein profiles (Figure S1C). Volcano plot analysis revealed 212 proteins that were significantly differentially expressed, consisting of 104 upregulated proteins and 108 downregulated proteins in HFD‐fed *ApoE^−/−^* mice relative to *ApoE^−/−^* control mice (Figure 1A). The top six significant DEPs were upregulated in atherosclerotic aorta, including Lgals3/galectin‐3, Nucb2, lghm, Vcam1, Serpina1e and Serpina3n (Figure 1B). We then performed KEGG analysis using these 212 DEPs. The result of KEGG revealed that these DEPs were mainly associated with the top 20 most altered pathways, including apoptosis, TNF signalling pathway, lysosome, phagosome and leukocyte transendothelial migration (Figure 1C). Consistently, gene set enrichment analysis (GSEA) suggested that the DEPs related to foal adhesion, leukocyte transendothelial migration, natural killer cell‐mediated cytotoxicity, Toll‐like receptor signalling pathway, and apoptosis were upregulated (Figure 1D). Furthermore, we also noticed that biological processes related to DEPs comprised regulation of cell killing, leukocyte chemotaxis, leukocyte migration, leukocyte migration and intrinsic apoptotic signalling (Figure S1D).

### 3.2 DEGs are identified by bioinformatic assays of mice atherosclerotic database

The gene expression data GSE83112, which comprised three *ApoE^−/−^* mice fed an ND (GSM2193157, GSM2193158 and GSM2193159) and three *ApoE^−/−^* mice fed an HFD (GSM2193160, GSM2193161 and GSM2193162). We performed a bioinformatic assay to investigate changes in the transcriptome of the aortas of HFD‐fed *ApoE^−/−^* mice compared to *ApoE^−/−^* control mice. We calculated Pearson correlation coefficient from the peak height to evaluate the correlation between these samples. Highly correlated samples had a quality value approaching 1, indicating that the preparation process remained largely stable and that the quality of the data was extremely high (Figure S1E). Principal component analysis demonstrated that atherosclerotic samples were clustered together, with two distinct groups clearly separated (Figure S1F). The heatmap showed there were 314 DEGs between normal and atherosclerosis. Of these 314 genes, 225 genes were upregulated, and 89 were downregulated in HFD‐fed *ApoE^−/−^* mice compared to *ApoE^−/−^* mice (Figure S1G). The criteria for identifying DEGs were established as *p*‐value below.05 and a Log 2 FoldChange ≥1, and a volcano plot was generated to represent the expression distribution of the DEGs (Figure 1E). KEGG enrichment analysis identified apoptosis as the predominant cell death form in atherosclerotic vascular cells (Figure 1F). There were 104 DEPs obtained from proteomic analysis of the aortic samples and 255 DEGs obtained from bioinformatic assay of the gene expression data GSE83112. We overlapped DEPs and DEGs using a Venn diagram, resulting in three cooperative DEPs and DEGs between normal and atherosclerosis (Figure 1G). These three coordinately and differentially expressed genes and proteins were upregulated in atherosclerotic aorta, including Lgals3, Vcam1 and Ctss (Figure 1H).

### 3.3 Human carotid database is clustered according to the PANoptosis‐related genes

**FIGURE 2:**
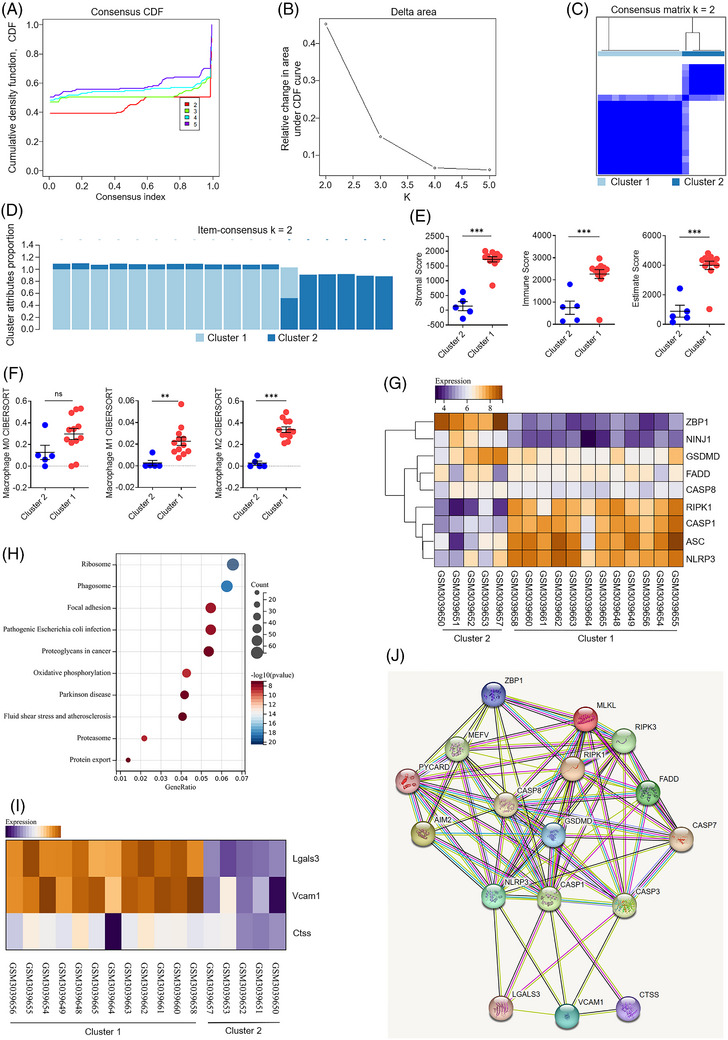
Molecular classification of human carotid atherosclerotic database based on PANoptosis‐related genes. (A) The CDF curves of consensus matrix for *k* = 2–5 are illustrated using distinct colours. *K* value represented the number of clusters. (B) The line graph of CDF area under curve is depicted at *k* = 2–5. The curve area with minimal variation is between *k* = 2 and *k* = 1, thus the clustering effect is relatively stable when *k* = 2. (C) Given that consensus matrix with *k* = 2 is an optimal choice, the entire cohort is separated into two clusters. (D) Item consensus plot when *k* = 2 indicates that the cluster pattern shared the acceptable level of purity in both clusters. (E) The distribution of immune, stromal and overall ESTIMATE scores is inferred by ESTIMATE algorithm between two clusters in the GSE111782 cohort, and three kinds of scores are substantially greater in Cluster 1 than Cluster 2. (F) The infiltration abundance of macrophage subsets is evaluated by CIBERSORT algorithm for two clusters, and Cluster 1 had a greater proportion of macrophage M1 and M2 than Cluster 2. (G) Landscape of PANoptosis‐related gene expression is depicted in Cluster 1 and Cluster 2. (H) KEGG enrichment of all DEGs shows the top 10 signalling pathways. The colour and size of each bubble indicate *p*‐value and gene count, respectively. (I) Landscape of three overlapping DEGs and DEPs, including Lgals3/galectin‐3, Vcam1 and Ctss is depicted in Cluster 1 and Cluster 2. Three key DEGs and DEPs are predicted to be activated in Cluster 1 compared to Cluster 2. (J) A PPI network of the three DEGs and DEPs, and 11 PANoptosis‐related genes is created according to the STRING database.

The regulation of PANoptosis has been associated with various infectious conditions, inflammatory diseases and cancers. PANoptosis‐like death (pyroptosis, apoptosis and necroptosis) occurs concurrently, and possesses interaction and co‐regulation during atherosclerosis. The GSE111782 dataset, which comprises the 18 human carotid endarterectomy specimens, was sourced from the GEO database. Then, we further performed a consensus clustering analysis and conducted molecular classification of GSE111782 based on the expression of PANoptosis‐related genes, including ZBP1, RIPK1, RIPK3, FADD, CASP8, NLRP3, ASC, CASP1, GSDMD, MLKL and NINJ1. All specimens were initially classified into *k* clusters ranging from two to five, and the cumulative distribution function (CDF) curves represented that *k* value was the optimal number when the CDF decline was not so pronounced (Figure 2A). The area under the CDF curve exhibited no obvious change when comparing the optimal *k* value with that of *k* − 1 (Figure 2B). The boundary between the two clusters of the samples became apparent when *k* = 2, so the atherosclerotic samples were categorised into Cluster 1 and Cluster 2 (Figure 2C). The item consensus plot revealed that 18 specimens were primarily categorised into two clusters (Cluster 1 with 12 specimens and Cluster 2 with five specimens), and one specimen was excluded due to the potential ambiguity (Figure 2D). The total scores for infiltrating stromal cells and total immune cells in two clusters were computed using the ESTIMATE algorithm. The two consensus clusters exhibited notable differences in immune, stromal and overall ESTIMATE scores, with Cluster 1 having a significantly higher score than Cluster 2 (Figure 2E). This study further investigated the infiltration abundance of macrophage subsets utilising the CIBERSORT algorithm. Macrophage M0 did not statistically differ between Cluster 1 and Cluster 2. In contrast, macrophage M1 and M2 were markedly more abundant in Cluster 1 in comparison with Cluster 2 (Figure 2F). Thus, we explained Cluster 1 as stromal‐hot and immune‐hot plaques with increasing macrophage infiltration, whereas Cluster 2 means stromal‐cold and immune‐cold plaques with reduced macrophage content. The expression patterns of 11 PANoptosis‐related genes were projected in Cluster 1 and Cluster 2, respectively (Figure 2G). Particularly, the expression levels of RIPK3, CASP1, ASC and NLRP3 were substantially elevated in Cluster 1 than in Cluster 2, while the expression levels of CASP8, FADD, GSDMD, NINJ1 and ZBP1 were significantly reduced in Cluster 1 than in Cluster 2 (Figure 2G). A total of 312 DEGs were identified between Cluster 1 and Cluster 2 utilising the limma program. We utilised these DEGs to conduct the functional enrichment analysis. KEGG analysis suggested that DEGs were focused mainly on the signalling pathways of fluid shear stress and atherosclerosis (Figure 2H). Notably, hierarchical clustering analysis demonstrated that the expression levels of Lgals3, Vcam1 and Ctss, identified as the co‐DEPs and DEGs, exhibited a significantly greater increase in cluster than in Cluster 2 (Figure 2I). The three co‐DEPs and DEGs, and 11 PANoptosis‐related genes were submitted to the STRING database, and PPI networks were established to assess the interactions among the candidates of co‐DEPs and DEGs and PANoptosis‐related genes (Figure 2J). Collectively, three co‐DEPs and DEGs, such as Lgals3, Vcam1 and Ctss, may serve as key regulatory factors for PANoptosis during atherosclerosis.

### 3.4 Galectin‐3 expression is increased in human and mouse atherosclerotic lesions

**FIGURE 3:**
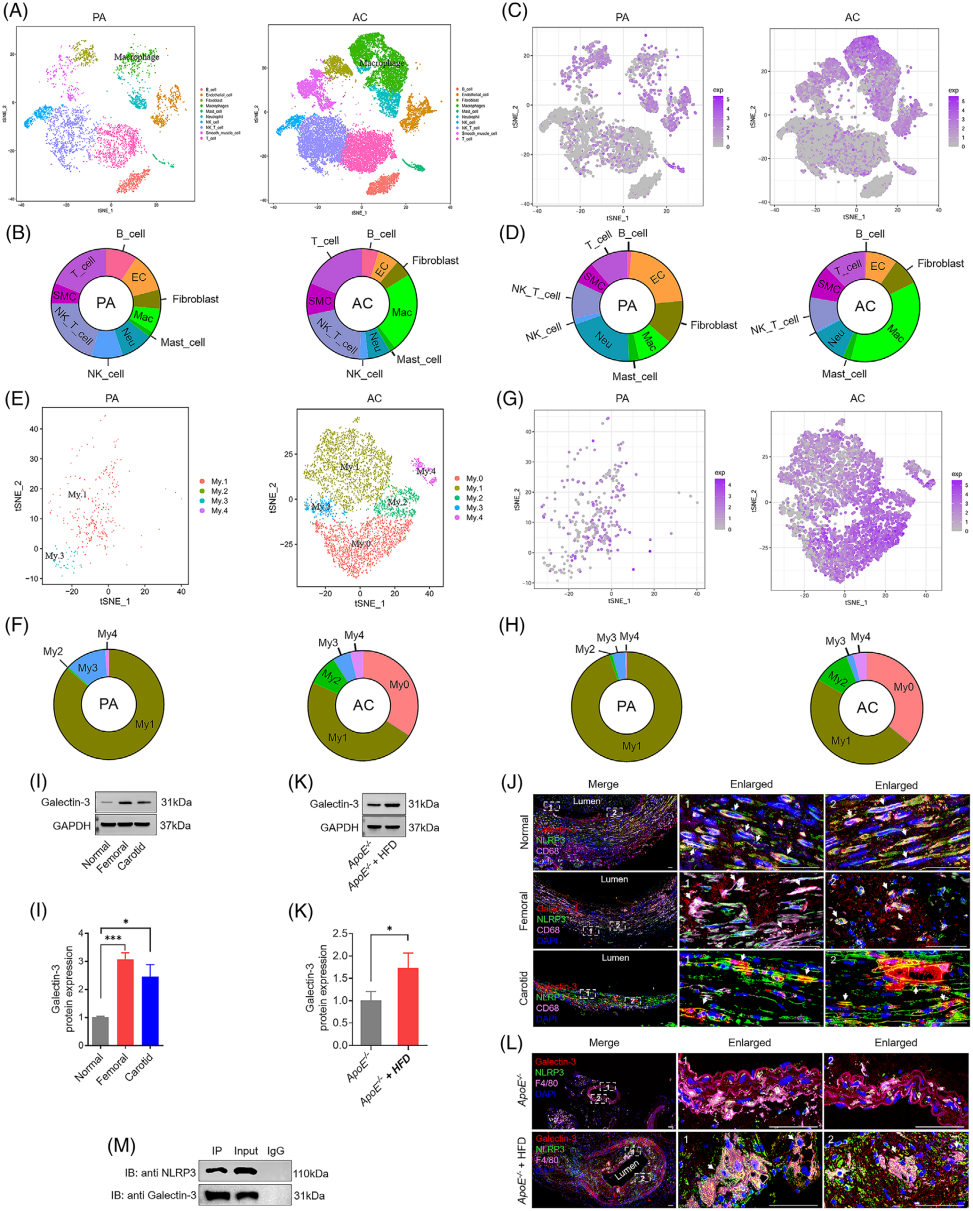
Galectin‐3 expression is abundant in human and mouse atherosclerotic lesions. (A) Ten main cell types are visualised in atherosclerotic core (AC) and proximal adjacent (PA) tissues by tSNE (t‐distributed stochastic neighbour embedding). (B) The macrophage population significantly increased in AC relative to PA. (C) Biaxial scatter plots show the expression pattern of galectin‐3 in total cell types between AC and PA. The colour scale represents expression levels in biaxial scatter plots (grey: low; pink: high). (D) Galectin‐3‐positive macrophages expanded in AC in comparison with PA. (E) Five macrophage subtypes are visualised in AC and PA tissues by tSNE. (F) *My.0* and *My.1* account for 34.1% and 47.6% of macrophages in AC, respectively. *My.2* significantly increased in AC relative to PA. (G) Biaxial scatter plots exhibit the expression pattern of galectin‐3 in macrophage subtypes between AC and PA. (H) Galectin‐3‐positive *My.0* and *My.1* account for 35.8% and 47.5% of galectin‐3‐positive macrophages in AC, respectively. Galectin‐3‐positive *My.2* expands in AC in comparison with PA. (I) Representative Western blots and relative quantitative analysis of galectin‐3 in human atherosclerotic lesions and peripheral normal artery. (J) Triple immunofluorescence staining for galectin‐3 (red), NLRP3 (green), CD68 (pink) and DAPI (blue) in human atherosclerosis and peripheral normal artery reveals the colocalisation of galectin‐3 and NLRP3 in CD68‐positive macrophages. Scale bar: 50 µm. (K) Representative Western blots and relative quantitative analysis of galectin‐3 in the aortas of *ApoE^−/−^* mice fed with an HFD or normal diet. (L) Triple immunofluorescence staining for galectin‐3 (red), NLRP3 (green), CD68 (pink) and DAPI (blue) in human atherosclerosis and peripheral normal artery reveals that galectin‐3 and NLRP3 are colocalised in CD68‐positive macrophages. Scale bar: 50 µm. (M) Cell lysates from ox‐LDL‐treated macrophages are immuno‐precipitated with anti‐galectin‐3 or anti‐NLRP3 antibodies, and blotted with anti‐NLRP3 or anti‐galectin‐3 antibodies. Data are derived from three to five independent experiments. ^*^ *p* ˂.05, ^**^ *p* ˂.01, ^***^p ˂.001 by Student's *t* test. ns: not significant.

To gain insight into the changing profile of cellular distribution and the cellular localisation of galectin‐3, we re‐analysed publicly available scRNA‐seq dataset on carotid endarterectomy tissue obtained from three patients. Distinct cell populations were profiled by *t*‐Distributed Stochastic Neighbour Embedding (tSNE). The 10 distinct cell types were visualised, including B cells, endothelial cells, fibroblasts, macrophages, mast cells, neutrophils, natural killer (NK) cells, NK T cells, smooth muscle cells and T cells (Figure 3A). The top 10 most significant markers were identified for each cluster relative to all other clusters, and these gene expression profiles were plotted using a heatmap (Figure S2A). Violin plot of signature genes confirmed the identities of 10 clusters (Figure S2B). The canonical macrophage markers, including HLA‐DRA (encoding β chain of MHC II), HLA‐DRB1 (encoding β chain of MHC II), HLA‐DPA1, HLA‐DPB1, HLA‐DQA1, C1Q1, C1QC, C1QB, CCL3 (encoding MIP‐1α) and SELENOP, were highly expressed in macrophage clusters as visualised by feature plots of tSNE (Figure S2C). Expression of 10 macrophage marker genes for 10 clusters was visualised by a violin plot (Figure S2D) and a dot plot (Figure S2E).

The percentage of macrophages was markedly elevated in the atherosclerotic cores (AC) group than in the proximal artery (PA) group (72.8% vs. 23.6% of total cells) (Figure 3B). We then analysed the expression pattern of galectin‐3 among the different cell types in the endarterectomy samples. As shown in biaxial scatter plots illustrating the expression patterns of galectin‐3 in different cell types (Figure 3C), the galectin‐3 positivity in macrophages was significantly greater in the AC group than in PA group (36.3% vs. 10.3% of total galectin‐3‐positive cells) (Figure 3D). Within the macrophages, we identified five subtypes (*My.0–My.4*) visualised with tSNE (Figure 3E) according to the well‐established marker genes in previously published papers.^24^ Macrophages were assigned into five subtypes by assessing marker genes visualised by a heatmap of the top 10 marker genes in each subtype (Figure S2F). The violin plot showed representative marker genes for five macrophage subtypes (Figure S2G). *My.0* exhibited the characteristics typically associated with transendothelial migration of recently recruited macrophages. *My.1* indicated inflammatory and/or tissue resident‐like macrophages. *My.2* showed the signs of Trem2‐expressing foamy macrophages. *My.3* resembled the features of dendritic cell phenotype. *My.4* was marked by regulatory T cells. Together, *My.0, My.1* and *My.2* most likely showed different phenotypes and macrophage activation states.^24^ Among them, *My.0* transmigrated from the bloodstream into AC through the endothelium and accounted for 34.1% of the proportion among macrophages, and the proportion of *My.2* was markedly higher in the AC group than in the PA group (9.1% vs.2% of macrophages) (Figure 3F). As shown in biaxial scatter plots illustrating the expression patterns of galectin‐3 in macrophage subtypes (Figure 3G), the galectin‐3‐positive *My.0* was exclusively identified in AC group and accounted for 35.8% of all galectin‐3‐positive macrophages, and the galectin‐3‐positive *My.2* was markedly elevated in AC group relative to PA group (10.6% vs.9% of all galectin‐3‐positive macrophages) (Figure 3H). In addition, the protein expression of galectin‐3 was significantly higher in human atheroma plaques than in histologically normal arteries (Figure 3I). Colocalisation assays using immunofluorescence further verified that galectin‐3/NLRP3 colocalised with CD68, a representative marker of macrophages (Figure 3J). The Pearson correlation coefficients (PCCs) for galectin‐3/NLRP3, galectin‐3/CD68 and NLRP3/CD68 were significantly elevated in human atheromatous plaques relative to histologically normal arterial tissue (Figure S3A). The protein expression of galectin‐3 was elevated in the aorta of HFD‐fed *ApoE^−/−^* mice in comparison with *ApoE^−/−^* control mice (Figure 3K). Triple immunofluorescence staining showed that galectin‐3 and NLRP3 were co‐expressed in F4/80‐positive macrophages in sections of Figure 3L. The PCC values of galectin‐3 with NLRP3, galectin‐3 with F4/80 and NLRP3 with F4/80 were statistically higher in the aorta of HFD‐fed *ApoE^−/−^* mice than in those of control *ApoE^−/−^* mice (Figure S3B). The binding of galectin‐3 to NLRP3 was confirmed by co‐immunoprecipitation assay using lysates from ox‐LDL‐treated macrophages (Figure 3M). Taken together, these data strongly indicated that atherosclerotic lesions were associated with the accumulation of macrophages and galectin‐3, principally expressed in atherosclerotic macrophages (especially inflammatory and foamy phenotypes), and might have interacted with NLRP3.

### 3.5 Pyroptosis, apoptosis and necroptosis in atherosclerotic lesions are potentially linked to the TLR4/MyD88/NF‐κB pathways

**FIGURE 4:**
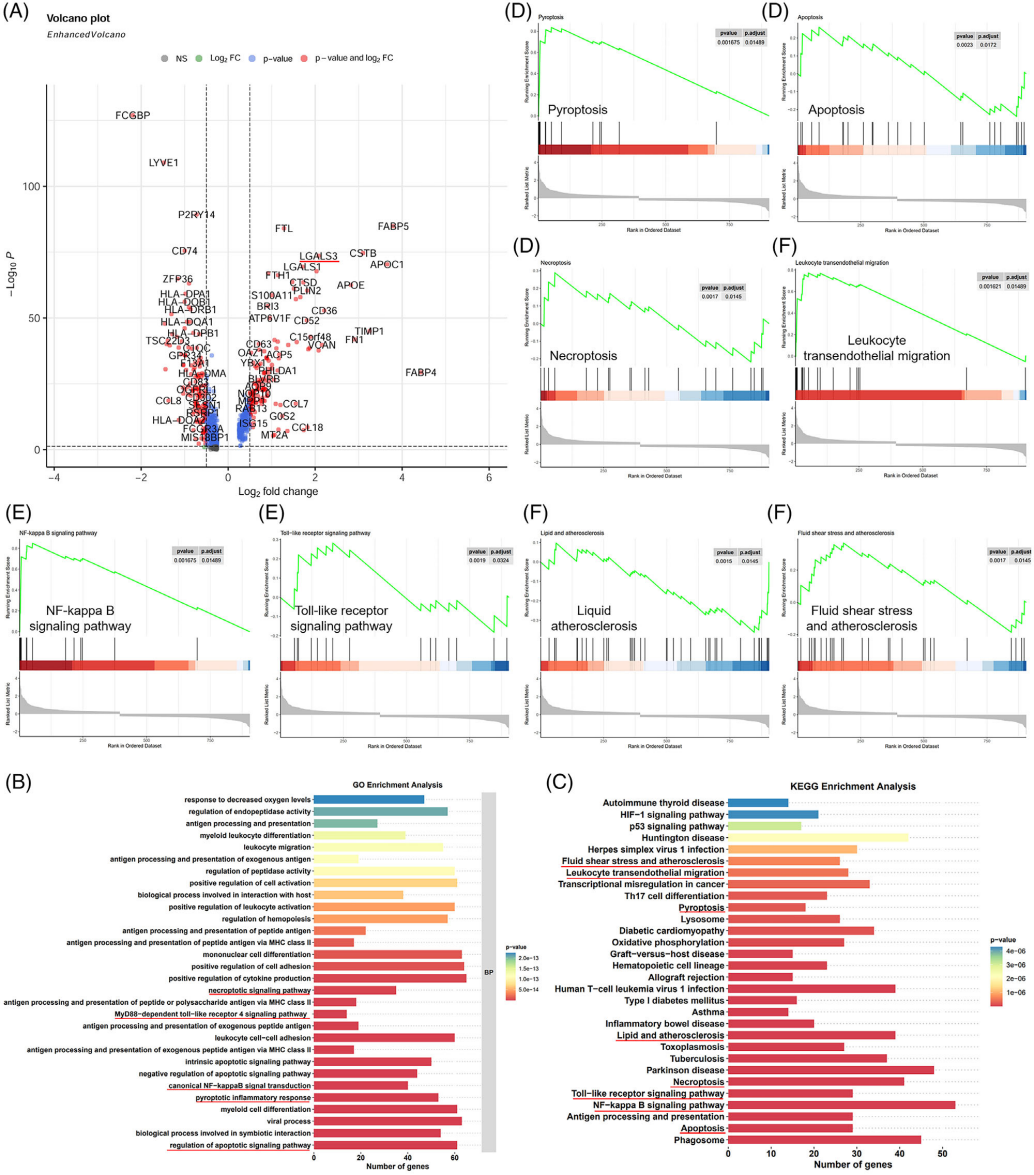
Gene Oncology (GO), Kyoto Encyclopedia of Genes and Genomes (KEGG), and gene set enrichment analysis (GSEA) of differentially expressed genes (DEGs) in macrophage between atherosclerotic core (AC) and proximal adjacent (PA) tissues. (A) Volcano plot of DEGs in macrophages is conducted between PA and AC. Lgals3/galectin‐3 are identified as a critical DEG expressed in macrophages. (B) Enriched GO terms are depicted with DEGs in macrophages, including necroptotic signalling pathway, pyroptotic inflammatory response, regulation of apoptotic signalling pathway, MyD88‐dependent Toll‐like receptor 4 signalling pathway, and canonical NF‐kappyB signal transduction, and so forth. (C) KEGG analysis is conducted using DEGs in macrophages, including fluid shear stress and atherosclerosis, leukocyte transendothelial migration, pyroptosis, lipid and atherosclerosis, necroptosis, Toll‐like receptor signalling pathway, NF‐kB signalling, and apoptosis. The *x*‐axis corresponds to the number of enriched DEGs, and the *y*‐axis corresponds to the enriched pathway. Colours indicate the *p*‐values, with red more significant enrichment. (D–F) Gene set of pyroptosis, apoptosis, necroptosis, NF‐kB signalling pathway, Toll‐like receptor signalling pathway, leukocyte transendothelial migration, lipid and atherosclerosis, and fluid shear stress and atherosclerosis are significantly upregulated in macrophages during atherosclerosis.

The DEGs in macrophage populations were illuminated in a volcano plot, which included Lgals3/galectin‐3 (Figure 4A). Macrophages are critical for the development and progression of atherosclerotic plaques. Here, GO‐BP and KEGG enrichment analysis were conducted on the DEGs in macrophages. GO‐BP and pathway enrichment analysis of DEGs revealed that macrophages experienced multiple biological processes related to pyroptosis, apoptosis and necroptosis and were implicated in the MyD88‐dependent Toll‐like receptor 4 signalling pathway and canonical NF‐kappa B signal transduction (Figure 4B). KEGG analysis indicated that gene sets were enriched in lipid and atherosclerosis, leukocyte transendothelial migration, apoptosis, necroptosis, pyroptosis, Toll‐like receptor and NF‐κB signalling pathway (Figure 4C).

Consistently, gene set enrichment analysis (GSEA) confirmed that genes related to necroptosis, pyroptosis and apoptosis were upregulated in atherosclerotic macrophages (Figure 4D). Furthermore, we also noticed that NF‐κB and Toll‐like receptor signalling pathways were significantly enriched in AC relative to PA (Figure 4E). In addition, numerous genes related to leukocyte transendothelial migration, liquid atherosclerosis, fluid shear stress and atherosclerosis were upregulated in AC but not in PA, suggesting activation of these pathways during atherosclerosis (Figure 4F). Taken together, these results indicate that the occurrence of necroptosis, pyroptosis and apoptosis may be associated with, but is not necessarily regulated by, the Toll‐like receptor and NF‐κB signalling pathways.

### 3.6 PANoptosis‐like cell death is activated in human atherosclerotic lesions

**FIGURE 5:**
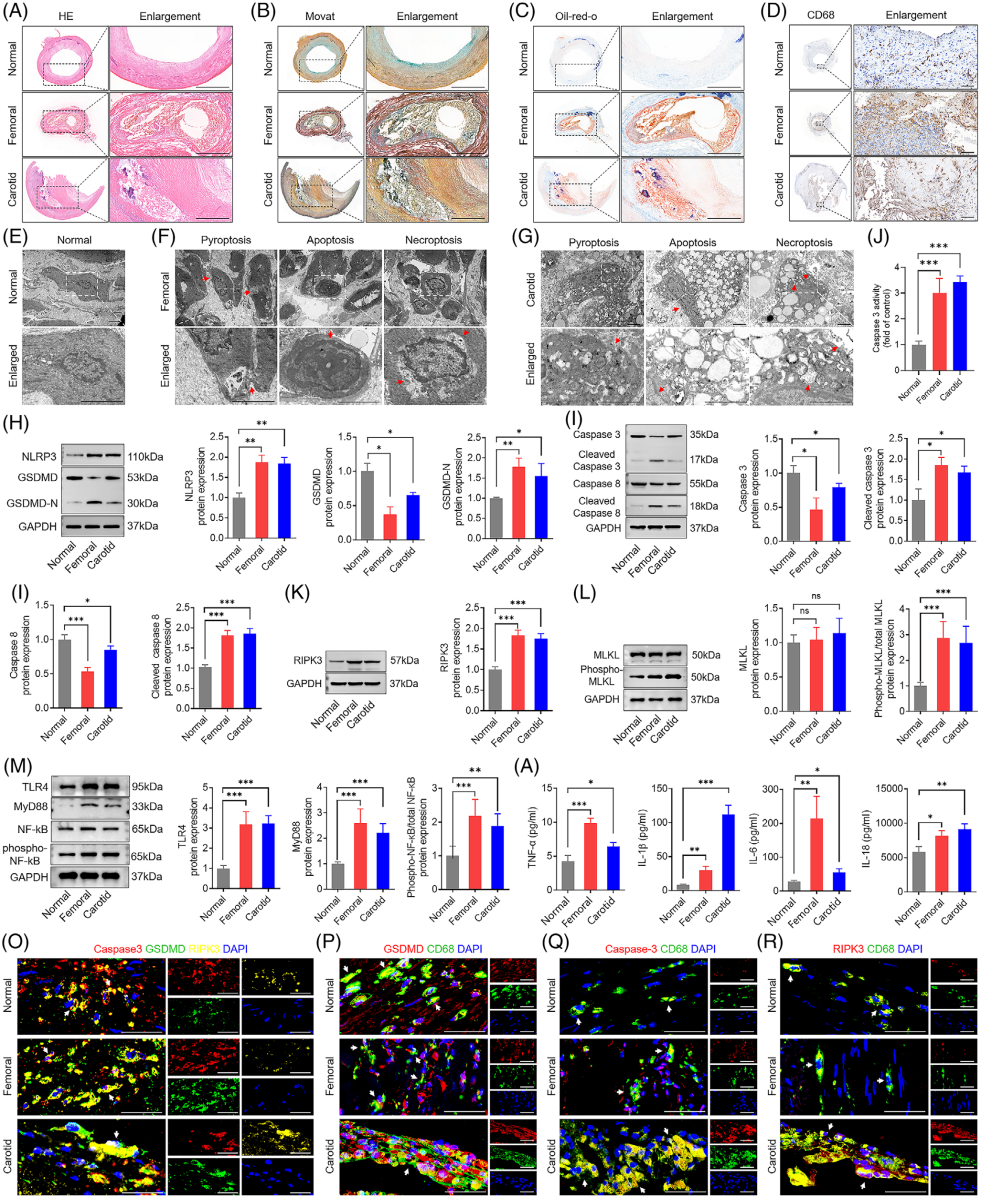
Pyroptosis, apoptosis and necroptosis in macrophages concurrently exist in human atherosclerotic lesions. (A–C) Necrotic core formation, lipid deposition and extracellular fibrosis are observed in lower extremity and carotid atherosclerotic lesions, but not in histologically normal artery by means of HE, Oil Red O, and Movat's staining. Scale bar: 1000 µm. (D) Immunohistochemical staining for CD68, a marker for macrophages, is conducted on histologically normal arteries as well as on lower extremity and carotid atherosclerotic lesions. Scale bar: 100 µm. (E–G) Compared with histologically normal artery (E), lower extremity (F) and carotid atherosclerotic lesions (G) show the pyroptotic, apoptotic and necroptotic characteristics of macrophages, including discontinuity of plasma membrane (red arrows), chromatin condensation, and electron‐light zones in transmission electron microscopy images. Scale bar: 2.5 µm. (H) Representative Western blots and relative quantitative analysis of NLRP3, GSDMD and GSDMD‐N in human atherosclerotic lesions and peripheral normal artery. (I) Representative Western blots and relative quantitative analysis of caspase‐3, cleaved caspase‐3, caspase‐8 and cleaved caspase‐8 in human atherosclerotic lesions and peripheral normal artery. (J) The capacity of caspase‐3 in human atherosclerotic lesions and peripheral normal artery. (K and L) Representative Western blots and relative quantitative analysis of RIPK3, MLKL and phospho‐MLKL in human atherosclerotic lesions and peripheral normal artery. (M) Representative Western blots and relative quantitative analysis of TLR4, MyD88 and phospho‐NF‐kB in human atherosclerotic lesions and peripheral normal artery. (N) Human atherosclerotic lesions release a significant amount of TNF‐1α, IL‐1β, IL‐18 and IL‐6, whereas the peripheral normal artery releases less. (O) Triple immunofluorescence staining for GSDMD (green), caspase‐3 (red), RIPK3 (yellow) and DAPI (blue) in human atherosclerosis and peripheral normal artery reveals the co‐existence of pyroptosis, apoptosis and necroptosis as evidenced by the colocalisation of GSDMD, caspase‐3 and RIPK3. Triple‐positive cells are shown by the arrows. Scale bar: 50 µm. (P–R) Double immunofluorescence staining for GSDMD (M)/caspase‐3 (N)/RIPK3 (O) (red), CD68 (green), and DAPI (blue) in human atherosclerosis and peripheral normal artery demonstrates the presence of pyroptotic, apoptotic and necroptotic markers in macrophages, as indicated by the colocalisation of caspase‐3/GSDMD/RIPK3 and CD68 (a macrophage marker). Double‐positive cells are shown by the arrows. Scale bar: 50 µm. Data are derived from three to five independent experiments. ^*^ *p* ˂.05, ^**^ *p* ˂.01, ^***^p ˂.001 by Student's *t* test. ns: not significant.

Pathologic intimal thickening and large fibroatheroma with necrotic cores were observed in conjunction with arterial stenosis by HE and Movat's staining (Figure 5A,B). Meanwhile, Oil Red O staining exhibited distinct lipid deposits in the core region of atherosclerotic fibrous plaques (Figure 5C). Atherosclerosis demonstrated the elevated CD68 expression in comparison to control vessels, as evidenced by immunohistochemistry (Figure 5D). Under the transmission electron microscope, the presence of lipid globules and the lack of contractile elements serve as distinguishing features of macrophages. Necroptosis is represented by cellular swelling, organelle enlargement, plasma membrane breakdown and subsequent release of intracellular components. Pyroptotic macrophages were identified by membrane thickening, abundant lysosomes, and formation of plasma membrane pores in human atherosclerotic lesions (Figure 5E–G). Macrophages undergoing apoptosis displayed characteristic changes of nuclear chromatin condensation and loss of cell volume within human atherosclerotic lesions (Figure 5E–G). Cytoplasmic vacuolisation, translucent electron‐light cytoplasm, and damaged cytoplasmic membrane integrity were observed in human atherosclerotic lesions relative to normal peripheral arteries (Figure 5E–G). Together, these results indicate the presence of pyroptotic, apoptotic and necroptotic macrophages within the human atherosclerotic lesions.

GSDMD is cleaved to produce active GSDMD‐N fragments, which promote the formation of plasma membrane pores to trigger pyroptosis. The protein expressions of GSDMD‐N and NLRP3 were consistently increased, while that of GSDMD was decreased in human atheroma plaques compared to histologically normal arteries (Figure 5H). The apoptosis intrinsic pathway is strictly mediated by the initiation of caspase‐3 and caspase‐8 cleavage. The protein expressions of cleaved caspase‐3 and cleaved caspase‐8 were notably elevated, while those of caspase‐3 and caspase‐8 were decreased in human atheroma plaques compared to histologically normal arteries (Figure 5I). The activity of caspase‐3 was significantly increased in human atheroma plaques compared to histologically normal arteries (Figure 5J). Necroptosis is an alternative mode of regulated necrosis and is dependent on a signalling pathway involving RIPK3 and pMLKL. The protein expressions of RIPK3 and pMLKL were elevated in human atheroma plaques in comparison with histologically normal arteries (Figure 5K,L). The TLR4/MyD88/NF‐κB signalling pathway may play a crucial role in the pathogenesis of atherosclerosis through the stimulation of PANoptosis‐like cell death. The protein expressions of TLR4, MyD88 and pNF‐κB were markedly elevated in human atheroma plaques relative to histologically normal arteries (Figure 5M). Compared with histologically normal arteries, proinflammatory mediators such as TNF‐1α, IL‐6, IL‐1β and IL‐18 were significantly increased in human atheroma plaques (Figure 5N). Colocalisation assays using immunofluorescence indicated that caspase‐3, GSDMD and RIPK3 were identified in three fluorescent‐labelled staining territories, disclosing potential interconnections between apoptosis, pyroptosis and necroptosis (Figure 5O). The PCC values of caspase‐3/GSDMD, caspase‐3/RIPK3 and GSDMD/RIPK3 were significantly higher in human atheromatous plaques than in histologically normal arteries (Figure S4A). Double immunofluorescence staining of caspase‐3/GSDMD/RIPK3 and macrophage marker CD68 showed that caspase‐3/GSDMD/RIPK3 expressions were localised in CD68‐labelled macrophages, indicating apoptosis/pyroptosis/necroptosis occurred predominantly in macrophages (Figure 5P–R). The PCC values of GSDMD/CD68, caspase‐3/CD68 and RIPK3/CD68 were significantly elevated in human atheromatous plaques compared to histologically normal arteries (Figure S4B). Collectively, these results suggest that the executor proteins of PANoptosis‐like cell death (necroptosis, pyroptosis and apoptosis) are upregulated in human atherosclerosis and are accompanied by robust release of proinflammatory cytokines.

### 3.7 Ox‐LDL activates the galectin‐3/TLR4/MyD88/NF‐κB pathway and induces pyroptosis, apoptosis and necroptosis in macrophages

**FIGURE 6:**
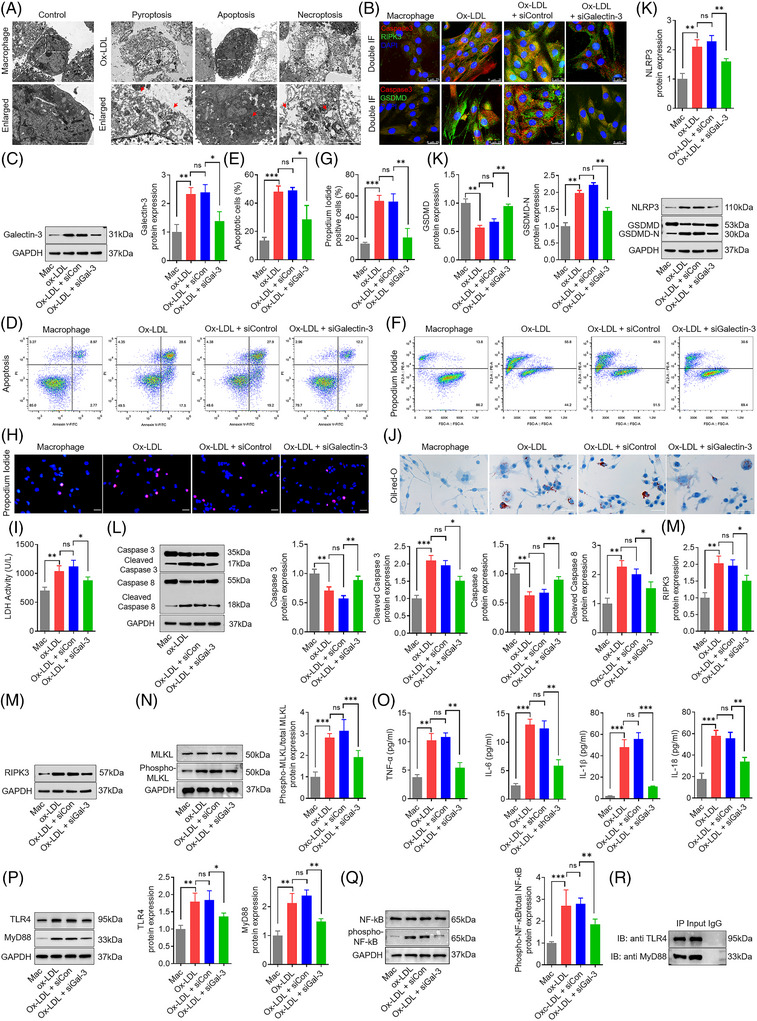
Silencing galectin‐3 downregulated TLR4/MyD88/NF‐kB expression and attenuated ox‐LDL induced pyroptotic, apoptotic, and necroptotic cell death in macrophages. (A) Electron microscopy ultrastructural analysis of control and ox‐LDL‐induced macrophages. Control macrophages have a normal‐looking cellular structure, whereas ox‐LDL‐induced macrophages show loss of cell plasma integrity, chromatin condensation or fragmentation, and electron‐light zone. Scale bar: 2.5 µm. (B) Confocal microscopy with double immunofluorescence staining for caspase‐3 (red) and RIPK3 (green) in macrophages show the colocalisation of apoptotic and necroptotic components. Confocal microscopy analysis of double immunofluorescence labelling is indicative of overlapping expression of caspase‐3 (red) and GSDMD (green) in macrophages. Scale bar: 25 µm. (C) Representative Western blots and relative quantitative analysis of galectin‐3 in control macrophages and cells treated with ox‐LDL, ox‐LDL plus siControl RNA, and ox‐LDL plus siGalectin‐3 RNA. (D and E) Flow cytometry (E) and quantification analysis (F) with annexin V/PI double staining show that ox‐LDL increased the percentage of apoptotic cells in macrophages, which is alleviated by silencing galectin‐3. (F–H) Flow cytometry (F) and quantification analysis (G) with PI/Hoechst staining (H) show that ox‐LDL enhanced PI uptake in macrophages, which is markedly blocked by silencing galectin‐3. Scale bar: 50 µm. (I) Silencing galectin‐3 abrogated LDH release in macrophages ignited by ox‐LDL. (J) Ox‐LDL induced the accumulation of intracellular lipid droplets in macrophages, which are potently reversed by silencing galectin‐3. Scale bar: 50 µm. (K) Representative Western blots and relative quantitative analysis of NLRP3, GSDMD and GSDMD‐N in control macrophages and cells treated with ox‐LDL, ox‐LDL plus siControl RNA, and ox‐LDL plus siGalectin‐3 RNA. (L) Representative Western blots and relative quantitative analysis of caspase‐3, cleaved caspase‐3, caspase‐8 and cleaved caspase‐8 in control macrophages and cells treated with ox‐LDL, ox‐LDL plus siControl RNA, and ox‐LDL plus siGalectin‐3 RNA. (M and N) Representative Western blots and relative quantitative analysis of RIPK3, MLKL and phospho‐MLKL in control macrophages and cells treated with ox‐LDL, ox‐LDL plus siControl RNA, and ox‐LDL plus siGalectin‐3 RNA. (O) Ox‐LDL treatment promotes the release of proinflammatory cytokines (TNF‐1α, IL‐1β, IL‐18 and IL‐6) from macrophages, which is markedly rescued by silencing galectin‐3. (P and Q) Representative Western blots and relative quantitative analysis of TLR4, MyD88, NF‐kB and phospho‐NF‐kB in control macrophages and cells treated with ox‐LDL, ox‐LDL plus siControl RNA, and ox‐LDL plus siGalectin‐3 RNA. (R) Cell lysates from ox‐LDL‐treated macrophages are immunoprecipitated with anti‐TLR4 or anti‐MyD88 antibodies, and blotted with anti‐TLR4 or anti‐MyD88 antibodies. Data are derived from three to five independent experiments. ^*^ *p* ˂.05, ^**^ *p* ˂.01, ^***^p ˂.001 by Student's *t* test. ns: not significant.

Transmission electron microscopy analysis reflected a dramatic rise in macrophage cell death with ultrastructural features of pyroptosis, apoptosis and necroptosis (Figure 6A). Confocal microscopy analysis of double immunofluorescence labelling showed that caspase‐3 colocalised with either RIPK3 or GSDMD, indicative of potential complex formation of apoptotic, necroptotic and pyroptotic proteins (Figure 6B). To further confirm that galectin‐3 induced programmed cell death, we silenced galectin‐3 expression in macrophages using siRNA. Incubation of macrophages with ox‐LDL led to the upregulation of galectin‐3, which was robustly depressed by silencing galectin‐3 (Figure 6C). Ox‐LDL treatment enlarged the proportion of apoptotic macrophages, and galectin‐3 silencing obviously reversed the pro‐apoptotic role of ox‐LDL on macrophages (Figure 6D,E). Seventy‐two hours of ox‐LDL treatment caused approximately 50% of macrophages to become PI‐positive, and galectin‐3 silencing markedly rescued the damaging effect of ox‐LDL on the cell plasma membrane (Figure 6F–H). Ox‐LDL enhanced lactate dehydrogenase (LDH) release in macrophages, which was potently abrogated by galectin‐3 silencing (Figure 6I). Macrophage foam cells are critical components of atherosclerotic plaques. Next, genetic knockdown of galectin‐3 ameliorated foam cell formation (Figure 6J).

To further elucidate the forms of cell death, we next assessed the pyroptotic, apoptotic and necroptotic markers by Western blotting. Pyroptotic cell death is mainly regulated by the NLRP3 inflammasome and proteolytic cleavage of GSDMD. Compared with unstimulated cells, ox‐LDL treatment elicited upregulation of NLRP3 and cleavage of GSDMD, and these were pronouncedly abrogated by genetic knockdown of galectin‐3 (Figure 6K). Cleaved caspase‐3 and caspase‐8 were used as hallmarks for apoptosis activation. The treatment of macrophages with ox‐LDL induced the cleaved activation of caspase‐3 and caspase‐8, which was inhibited by genetic knockdown of galectin‐3 (Figure 6L). Ox‐LDL treatment elevated the activity of caspase‐3, and these were mostly reversed by genetic knockdown of galectin‐3 (Figure S5A). Necroptosis activation was monitored by RIPK3 and pMLKL. Genetic knockdown of galectin‐3 in macrophages inhibited the ox‐LDL‐induced upregulation of RIPK3 and pMLKL (Figure 6M,N). Activation of the programmed macrophage death leads to the production of diverse cytokines. Ox‐LDL‐treated macrophages released a significant amount of TNF‐α, IL‐6, IL‐1β and IL‐18, and galectin‐3 genetic knockdown in macrophages attenuated ox‐LDL‐induced cytokine release (Figure 6O). Additionally, to clarify whether galectin‐3 activates the TLR4/MyD88/NF‐κB pathways in vitro, the expression levels of relevant proteins were assessed in four groups of macrophages by Western blotting. We found that ox‐LDL‐stimulated TLR4/MyD88/NF‐κB signalling activation was reversed by knockdown of galectin‐3 with siRNA (Figure 6P,Q). Collectively, these findings confirm that ox‐LDL treatment of macrophages induces the expression of the pyroptotic, apoptotic and necroptotic markers, promotes the release of proinflammatory cytokines, and activated the TLR4/MyD88/NF‐κB signalling axis, which is robustly abolished by genetic knockdown of galectin‐3.

NF‐κB is normally sequestered in the cytoplasm and translocates to the nucleus upon pathogen stimulation. Ox‐LDL treatment induced nuclear translocation of NF‐κB in macrophages, an effect that was markedly attenuated by genetic knockdown of galectin‐3 (Figure S5B). Our findings indicated that Ox‐LDL treatment induced PI‐positive cell death in macrophages. Next, we examined whether the PANoptosis‐like cell death following ox‐LDL treatment is primarily dependent on this kind of combined cell death. Therefore, pretreatment with various combinations of inhibitors for apoptosis (Z‐VAD), pyroptosis (DSF) and necroptosis (Nec‐1) was employed to evaluate the protective effects on cell loss against ox‐LDL treatment. The combination of Z‐VAD with DSF, with both Z‐VAD and Nec‐1, or with DSF and Nec‐1 had a better protective effect on PI‐positive cell death than Z‐VAD, DSF, or Nec‐1 alone (Figure S3A,B). Moreover, the triple combination of Nec‐1, Z‐VAD and DSF had the largest protective effect on PI‐positive cell death compared with each of the double combinations (Figure S5C,D). Collectively, these results show that ox‐LDL‐induced cell death is mainly driven by the coordination of pyroptosis, apoptosis and necroptosis.

### 3.8 Silencing galectin‐3 depresses ox‐LDL‐induced pyroptosis, apoptosis and necroptosis in macrophages, which are partially reversed by NLRP3 agonist

**FIGURE 7:**
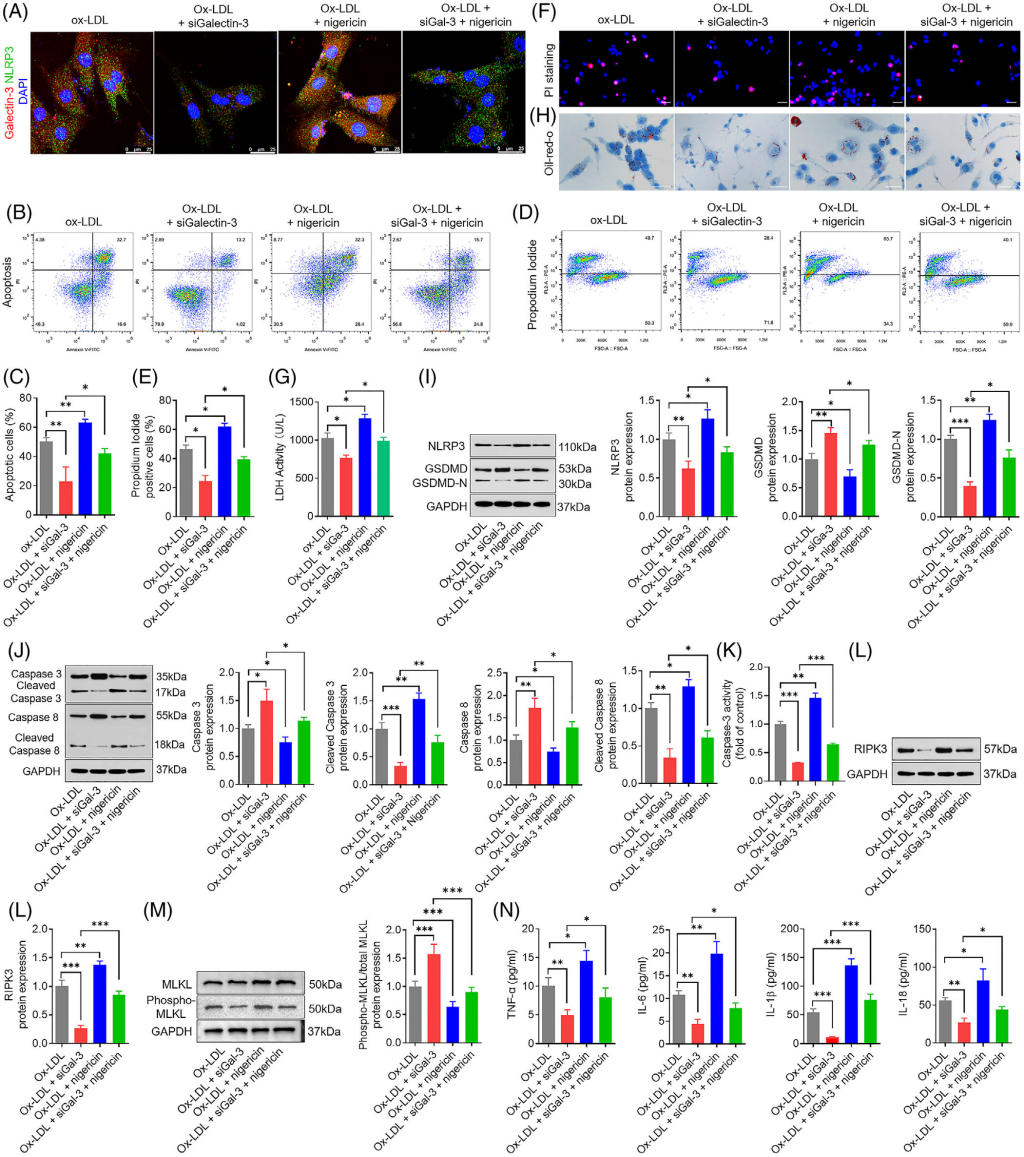
NLRP3 agonist nigericin counteracted the inhibitory effect of silencing galectin‐3 on pyroptosis, apoptosis and necroptosis in macrophages. (A) Confocal microscopy with double immunofluorescence staining for galectin‐3 (red) and NLRP3 (green) in macrophages reveals the colocalisation of galectin‐3 with NLRP3. Scale bar: 25 µm. (B and C) Flow cytometry (B) and quantification analysis (C) with annexin V/PI double staining show that silencing galectin‐3 decreases the percentage of apoptotic cells in ox‐LDL‐induced macrophages, and nigericin robustly blunts the inhibitory effect of siGalectin‐3. (D–F) Flow cytometry (D) and quantification analysis (E) with PI/Hoechst staining (F) show that silencing galectin‐3 diminishes the percentage of PI‐positive cells in ox‐LDL‐induced macrophages, and nigericin mostly abolishes the protective effect of siGalectin‐3. Scale bar: 50 µm. (G) Silencing galectin‐3 suppresses the LDH release in ox‐LDL‐induced macrophages, which is largely abrogated by nigericin. (H) Silencing galectin‐3 lessens the intracellular lipid droplet in ox‐LDL‐induced macrophages, while nigericin exerts the opposite effect. Scale bar: 50 µm. (I) Representative Western blots and relative quantitative analysis of NLRP3, GSDMD and GSDMD‐N in macrophages treated with ox‐LDL, ox‐LDL plus galectin‐3 siRNA, ox‐LDL plus nigericin, and ox‐LDL plus galectin‐3 siRNA plus nigericin. (J) Representative Western blots and relative quantitative analysis of caspase‐3, cleaved caspase‐3, caspase‐8 and cleaved caspase‐8 in macrophages treated with ox‐LDL, ox‐LDL plus galectin‐3 siRNA, ox‐LDL plus nigericin, and ox‐LDL plus galectin‐3 siRNA plus nigericin. (K) The activity of caspase‐3 in macrophages treated with ox‐LDL, ox‐LDL plus galectin‐3 siRNA, ox‐LDL plus nigericin, and ox‐LDL plus galectin‐3 siRNA plus nigericin. (L and M) Representative Western blots and relative quantitative analysis of RIPK3, MLKL and phospho‐MLKL in macrophages treated with ox‐LDL, ox‐LDL plus galectin‐3 siRNA, ox‐LDL plus nigericin, and ox‐LDL plus galectin‐3 siRNA plus nigericin. (N) Silencing galectin‐3 inhibits the release of inflammatory cytokines (TNF‐1α, IL‐1β, IL‐18 and IL‐6) in ox‐LDL‐induced macrophages, and nigericin effectively blocks the role of siGalectin‐3. Data are derived from three to five independent experiments. ^*^ *p* ˂.05, ^**^ *p* ˂.01, ^***^p ˂.001 by Student's *t* test. ns: not significant.

To further explore the downstream targets of galectin‐3 in regulating pyroptosis, apoptosis and necroptosis, we evaluated the interaction between galectin‐3 and NLRP3. Co‐IP assay showed that the connection between galectin‐3 and NLRP3 was evident in cell lysates pulled down by either galectin‐3 or NLRP3 immunoprecipitation. Confocal microscopy analysis of double immunofluorescence labeling showed colocalisation of galectin‐3 with NLRP3, revealing the potential overlap between galectin‐3 and NLRP3 (Figure 7A).

To evaluate the impact of galectin‐3 and NLRP3 signalling pathway on the ox‐LDL‐mediated pyroptosis, apoptosis and necroptosis in macrophages, ox‐LDL‐treated macrophages were treated with galectin‐3 siRNA, NLRP3 agonist nigericin, and both together. In comparison with ox‐LDL‐treated macrophages, silencing of galectin‐3 with RNA interference led to a decrease in apoptotic cells, and nigericin markedly blunted this response (Figure 7B,C). Similarly, PI‐positive cells were markedly decreased by knockdown of galectin‐3 and, conversely, strongly enlarged in the presence of nigericin (Figure 7D–F). The inhibitory activity of galectin‐3 knockdown on PI uptake was disrupted by the addition of nigericin (Figure 7D–F). Similarly, the increase in LDH release by ox‐LDL treatment was significantly inhibited by galectin‐3 genetic knockdown and, conversely, further strengthened by the administration of nigericin (Figure 7G). The inhibitory effect of siGalectin‐3 on LDH release was likewise abolished by the addition of nigericin (Figure 7G). Macrophage‐derived foam cell formation represents a critical event in the development of atherosclerosis. Nigericin exacerbated the generation of macrophage foam cells and effectively counteracted the curative role of galectin‐3 genetic knockdown (Figure 7H).

Knockdown of galectin‐3 with siRNA reduced NLRP3 expression in ox‐LDL‐treated macrophages, whereas NLRP3 agonist nigericin enhanced its expression (Figure 7I). Downregulation of NLRP3 induced by silencing galectin‐3 was abolished by nigericin (Figure 7I). Silencing galectin‐3 depressed, conversely, nigericin enhanced the cleavage of GSDMD in ox‐LDL‐treated macrophages (Figure 7I). Nigericin abolished the inhibition of GSDMD cleavage caused by silencing galectin‐3 in ox‐LDL‐treated macrophages (Figure 7I). The protein expression levels of apoptosis‐associated factors in ox‐LDL‐treated macrophages were also affected by silencing galectin‐3, including caspase‐3 and caspase‐8. Compared with ox‐LDL‐induced macrophages, the inhibitory effect of galectin‐3 silencing on caspase‐3 and caspase‐8 cleavage was reversed in the knockdown of galectin‐3 and the nigericin group, and NLRP3 agonist nigericin alone aggravated caspase‐3 and caspase‐8 cleavage (Figure 7J). Silencing galectin‐3 with siRNA reduced caspase‐3 activity in ox‐LDL‐treated macrophages, whereas NLRP3 agonist nigericin enhanced its activity (Figure 7K). Depression of caspase‐3 activity induced by silencing galectin‐3 was reversed by the addition of nigericin (Figure 7K). Interestingly, when ox‐LDL‐induced macrophages were treated with both galectin‐3 siRNA and nigericin, the downregulation of RIPK3 and MLKL induced by silencing galectin‐3 was abolished in the presence of NLRP3 agonist nigericin (Figure 7L,M). Strikingly, the production of inflammatory mediators (TNF‐α, IL‐6, IL‐18 and IL‐1β) in ox‐LDL‐treated macrophages was reduced when galectin‐3 was silenced and, conversely, enhanced when NLRP3 agonist nigericin was added (Figure 7N). NLRP3 agonist nigericin disrupted the inhibitory effect of cytokine release induced by galectin‐3 silencing after ox‐LDL treatment (Figure 7N). Taken together, these results support that galectin‐3 initiates pyroptosis, apoptosis and necroptosis in macrophages, at least partially, by activating NLRP3.

To further probe the protective role of galectin‐3 in PANoptosis‐like cell death, TD139 was employed to inhibit the galectin‐3 expression in ox‐LDL‐induced macrophages (Figure S6A). The expression levels of pyroptosis, apoptosis and necroptosis‐related proteins in ox‐LDL‐treated macrophages were measured by Western blotting. After the inhibition of galectin‐3 by adding TD139, the protein expression of NLRP3 and the cleavage of GSDMD were markedly downregulated in ox‐LDL‐treated macrophages (Figure S6B). TD139 treatment significantly suppressed the cleavage of caspase‐3 and caspase‐8 induced by ox‐LDL treatment in macrophages (Figure S6C). Similarly, TD139 suppressed the expression of RIPK3 and pMLKL in ox‐LDL‐treated macrophages (Figure S6D). The above data suggest that galectin‐3 inhibition alleviates the occurrence of pyroptosis, apoptosis and necroptosis during atherosclerosis in vitro. DMSO was employed as the solvent for the NLRP3 agonist nigericin and the galectin‐3 inhibitor TD139 prior to their addition to the cells. The results show that DMSO alone does not affect the expression of pyroptosis, apoptosis and necroptosis‐related proteins in ox‐LDL‐induced macrophages.

### 3.9 Galectin‐3 deficiency attenuates, conversely, and NLRP3 agonist exacerbates the atherosclerotic pyroptosis, apoptosis and necroptosis in HFD‐fed *ApoE* ^−/−^ mice

**FIGURE 8:**
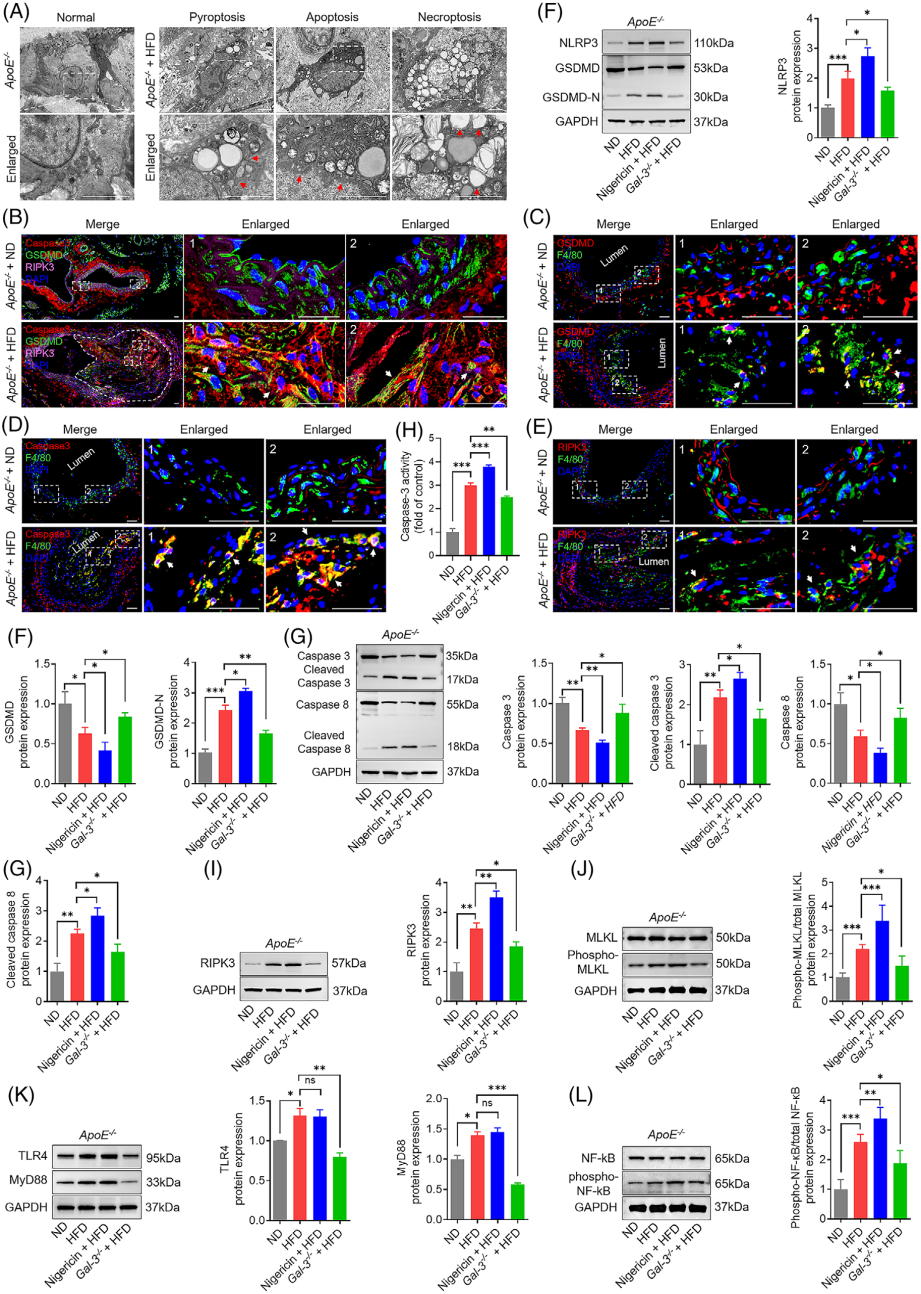
Pyroptosis, apoptosis and necroptosis in macrophages coordinately occurred in *ApoE^−/−^* mice fed an HFD, which are alleviated by galectin‐3 deficiency, and conversely are aggravated by NLRP3 agonist nigericin. (A) Pyroptosis, apoptosis and necroptosis of macrophages are identified in the aortas of *ApoE^−/−^* mice fed an HFD, as evidenced by plasma membrane pore (red arrows), chromatin condensation (red arrows), and electron‐light zone (red arrows) by transmission electron microscopy. Scale bar: 2.5 µm. (B) Triple immunofluorescence staining for GSDMD (green), caspase‐3 (red), RIPK3 (pink) and DAPI (blue) in the aortas of *ApoE^−/−^* mice fed HFD or normal diet reveals the potential crosstalk among pyroptosis, apoptosis and necroptosis as evidenced by the colocalisation of GSDMD, caspase‐3 and RIPK3. Three‐positive cells are shown by the arrows. Scale bar: 50 µm. (C–E) Dual immunofluorescence staining for caspase‐3 (C)/GSDMD (D)/RIPK3 (E) (red), F4/80 (green), and DAPI (blue) in the aortas of *ApoE^−/−^* mice fed an HFD or normal diet demonstrate that GSDMD/caspase‐3/RIPK3 immunoreactivity colocalises with macrophage marker CD68. Scale bar: 50 µm. (F) Representative Western blots and relative quantitative analysis of NLRP3, GSDMD and GSDMD‐N in the aortas of *ApoE^−/−^* mice fed with a normal diet or HFD, NLRP3 agonist nigericin‐treated *ApoE^−/−^* mice fed with an HFD, and *Galectin‐3^−/−^*/*ApoE^−/−^* mice fed with an HFD. (G) Representative Western blots and relative quantitative analysis of caspase‐3, cleaved caspase‐3, caspase 8 and cleaved caspase 8 in the aortas of *ApoE^−/−^* mice fed with a normal diet or HFD, nigericin‐treated *ApoE^−/−^* mice fed with HFD, and *Galectin‐3^−/−^*/*ApoE^−/−^* mice fed with HFD. (H) The activity of caspase‐3 in the aortas of *ApoE^−/−^* mice fed with a normal diet or HFD, nigericin‐treated *ApoE^−/−^* mice fed with HFD, and *Galectin‐3^−/−^*/*ApoE^−/−^* mice fed with an HFD. (I and J) Representative Western blots and relative quantitative analysis of RIPK3, MLKL and phospho‐MLKL in the aortas of *ApoE^−/−^* mice fed with a normal diet or HFD, nigericin‐treated *ApoE^−/−^* mice fed with HFD, and *Galectin‐3^−/−^*/*ApoE^−/−^* mice fed with an HFD. (K and L) Representative Western blots and relative quantitative analysis of TLR4, MyD88, NF‐κB and phospho‐NF‐κB in the aortas of *ApoE^−/−^* mice fed with a normal diet or HFD, nigericin‐treated *ApoE^−/−^* mice fed with HFD, and *Galectin‐3^−/−^*/*ApoE^−/−^* mice fed with an HFD. *n* = 4–8 mice per group. ^*^ *p* ˂.05, ^**^ *p* ˂.01, ^***^p ˂.001 by Student's *t* test. ns: not significant.

Atherosclerotic aortas from HFD‐fed *ApoE^−/−^* mice were analysed at the ultrastructural level using transmission electron microscopy. Apoptotic macrophages within atherosclerotic plaques were represented by cytoplasmic shrinkage, nuclear fragmentation and chromatin condensation (Figure 8A). Pyroptotic macrophages within atherosclerotic plaques showed cellular swelling and plasma membrane pore formation without concomitant apoptotic nuclear changes (Figure 8A). Macrophages undergoing necroptosis exhibited electron‐lucent cytoplasm, loss of plasma membrane integrity and massive vacuolisation (Figure 8A). Macrophage apoptosis, pyroptosis and necroptosis inside atherosclerotic lesions can be morphologically detected by transmission electron microscopy. Immunofluorescence colocalisation assays demonstrated that caspase 3, GSDMD and RIPK3 were co‐expressed in the aortas of HFD‐fed *ApoE^−/−^* mice, indicating crosstalk among apoptosis, pyroptosis and necroptosis (Figure 8B). The PCC values of caspase‐3/GSDMD, caspase‐3/RIPK3 and GSDMD/RIPK3 were significantly higher in the atherosclerotic aortas of HFD‐fed *ApoE^−/−^* mice compared with those in the aortas of *ApoE^−/−^* control mice (Figure S7A). Using double immunofluorescence microscopy, caspase‐3, GSDMD and RIPK expressions were each found to co‐localise with the F4/80‐positive area as seen on the overlay, indicating apoptosis, pyroptosis and necroptosis occurred predominantly in macrophages (Figure 8C–E). The PCC values of GSDMD with F4/80, caspase‐3 with CD68, and RIPK3 with CD68 were significantly elevated in the atherosclerotic aortas of HFD‐fed *ApoE^−/−^* mice compared to the aortas of *ApoE^−/−^* control mice (Figure S7B).

Given our observation that galectin‐3, a DEP identified by proteomics, was markedly upregulated in advanced atherosclerotic plaques of both human and mice, was enriched in carotid endarterectomy samples with the higher PANoptosis related gene profiles from GE111782, and directly bound to NLRP3 inflammasome to activate PANoptosis, we postulated that galectin‐3 ablation and NLRP3 activation would differentially impact the atherosclerotic pyroptosis, apoptosis and necropotosis in vivo. Hence, 4‐week *ApoE^−/−^* mice and *Galetin‐3^−/−^/ApoE^−/−^* mice were maintained on an HFD for 16 weeks, and NLRP3 agonist nigericin was simultaneously administered to *ApoE^−/−^* mice when feeding on an HFD. NLRP3 expression and GSDMD‐N cleavage were markedly increased in HFD‐fed *ApoE^−/−^* mice compared with those in *ApoE^−/−^* control mice (Figure 8F). Nigericin deteriorated, conversely, galectin‐3 deficiency attenuated the NLRP3 expression and GSDMD cleavage in HFD‐fed *ApoE* ^−/−^ mice (Figure 8F). HFD induced the cleavage of caspase‐3 and caspase‐8 in *ApoE^−/−^* mice (Figure 8G). The cleavage of caspase‐3 and caspase‐8 was pronouncedly deteriorated by nigericin and, conversely, substantially alleviated by galectin‐3 deficiency (Figure 8G). The activity of caspase‐3 was markedly elevated in HFD‐fed *ApoE^−/−^* mice compared to that in *ApoE^−/−^* control mice (Figure 8H). Nigericin enlarged, conversely, galectin‐3 deficiency depressed the caspase‐3 activity in HFD‐fed *ApoE* ^−/−^ mice (Figure 8H). Compared with *ApoE^−/−^* control mice, HFD‐fed *ApoE^−/−^* mice exhibited a substantial elevation of RIPK3 and pMLKL expression levels (Figure 8I,J). Strikingly, nigericin treatment enhanced the expression levels of RIPK3 and pMLKL, while RIPK3 and pMLKL expression levels were markedly decreased in *Galetin‐3^−/−^/ApoE^−/−^* mice than in *ApoE^−/−^* mice (Figure 8I,J). As prior analysis suggested, GO and KEGG analyses demonstrated that DEGs in macrophages were mainly concentrated in MyD88‐dependent Toll‐like receptor 4, Toll‐like receptor and NF‐κB signalling pathways. TLR4, MyD88 and phospho‐NF‐κB expression levels were relatively higher in HFD‐fed *ApoE^−/−^* mice than those in *ApoE^−/−^* mice (Figure 8K,L). The expression levels of TLR4, MyD88 and pNF‐κB did not alter after nigericin treatment in HFD‐fed *ApoE^−/−^* mice, but were substantially lower in *Galetin‐3^−/−^/ApoE^−/−^* mice fed on HFD (Figure 8K,L). Collectively, our data suggest that NLRP3 agonist nigericin exacerbates, conversely, galectin‐3 deficiency mitigates the atherosclerotic pyroptosis, apoptosis and necroptosis in HFD‐fed *ApoE^−/−^* mice. Mechanically, TLR4, MyD88 and pNF‐κB expressions were elevated in HFD‐fed *ApoE^−/−^* mice. Galectin‐3 deficiency depressed, conversely, NLRP3 agonist nigericin did not influence TLR4/MyD88/pNF‐κB pathway

### 3.10 Genetic knocking down of TLR4 reduces atherosclerotic lesions in HFD‐fed *ApoE* ^−/−^ mice via inhibiting the downstream MyD88/NF‐κB/NLRP3 signalling pathway

**FIGURE 9:**
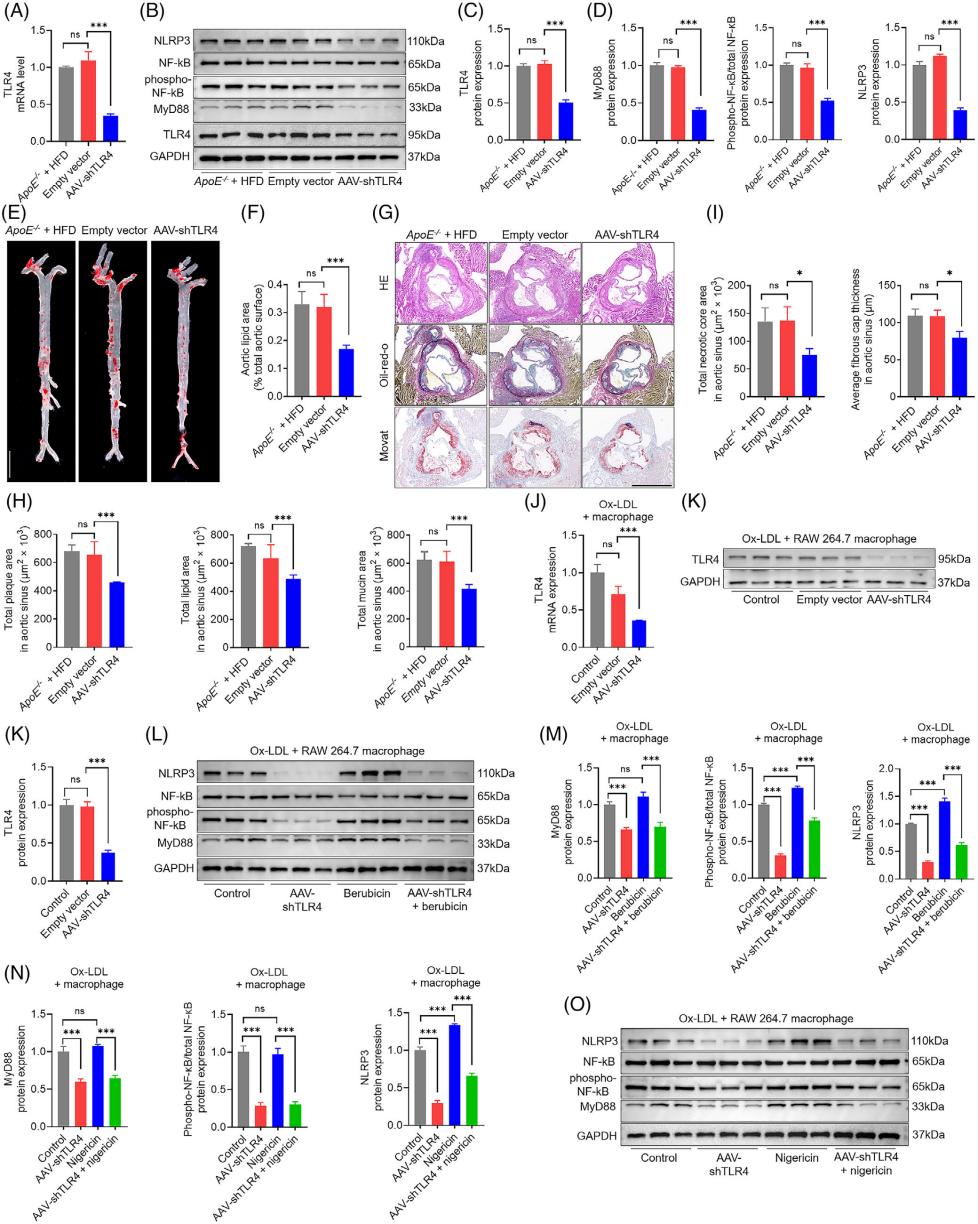
TLR4 silencing inhibits the development of atherosclerotic lesions via the downstream MyD88/NF‐kB/NLRP3 signalling molecules. (A) The mRNA level of TLR4 is markedly reduced in the atherosclerotic aorta of HFD‐fed ApoE*^−/−^* mice administered by AAV‐F4/80‐shTLR4. (B–D) Representative Western blots and relative quantitative analysis of TLR4, MyD88, NF‐kB, phospho‐NF‐kB and NLRP3 in the aortas of HFD‐fed ApoE−/− mice, empty vector‐treated HFD‐fed ApoE−/− mice and shTLR4‐treated HFD‐fed ApoE−/− mice. (E) Representative en face Oil Red O‐stained images of the entire aorta are obtained from HFD‐fed ApoE−/− mice, empty vector‐treated HFD‐fed ApoE−/− mice and shTLR4‐treated HFD‐fed ApoE−/− mice. Scale bar: 50 mm. (F) En face lesion area is quantified as a percentage of the total aortic surface area. After 16 weeks of HFD feeding, en face lesion areas are significantly smaller in HFD‐fed *ApoE^−/−^* mice treated with shTLR4 compared with those treated with the empty vector. (G) Representative sections of HE, Oil Red O and Movat's staining in the aortic sinuses are acquired from three different groups of mice. Scale bar: 1 mm. (H) Aortic sinus plaque lesion area, lipid lesion area and mucin area are represented as total area in µm^2^. Plaque area, lipid lesion area (red) and mucin area (blue‐green) are much smaller in HFD‐fed *ApoE^−/−^* mice treated with shTLR4 compared with those treated with empty vector. (I) Necrotic core area is calculated as the total area in µm^2^. Fibrous cap thickness is designated as average thickness in µm. TLR4 silencing diminishes the necrotic core area and fibrous cap thickness in vivo. (J and K) The silencing efficacy of TLR4 in ox‐LDL‐treated RAW 264.7 macrophages is verified by western blotting and RT‐ qPCR. (L and M) Representative Western blots and relative quantitative analysis of MyD88, NF‐kB, phospho‐NF‐kB and NLRP3 in ox‐LDL‐induced macrophages transduced by shTLR4 with or without co‐treatment by the NF‐κB activator berubicin. (N and O) Representative Western blots and relative quantitative analysis of MyD88, NF‐κB, phospho‐NF‐κB and NLRP3 in ox‐LDL‐induced macrophages transduced by shTLR4 with or without co‐treatment by the NLRP3 activator nigericin. *n* = 4–8 mice per group. Data are derived from three to five independent experiments. ^*^ *p* ˂.05, ^**^ *p* ˂.01, ^***^p ˂.001 by Student's *t* test. ns: not significant.

To further elucidate the role of TLR4/MyD88/NF‐κB/NLRP3 signalling pathway on atherosclerotic plaque development, 4‐week‐old *ApoE^−/−^* mice received, via tail vein injection, AAV encoding either macrophage‐specific shRNA targeting the TLR4 gene (driven by the F4/80 promoter; AAV‐F4/80‐shTLR4) or a control empty vector (AAV‐empty vector). These mice were maintained on an ND for a 2‐week period, followed by an atherogenic HFD for 16 weeks to induce atherosclerosis. TLR4 expression in aortic tissue was assessed after 16 weeks of HFD feeding, and both mRNA and protein levels were significantly reduced, as confirmed by RT‐qPCR and Western blotting, demonstrating the high in vivo efficacy of AAV‐F4/80‐shTLR4 in silencing TLR4 expression (Figure 9A–C). Downstream signalling molecules, including MyD88, pNF‐κB and NLRP3, were significantly downregulated following administration of AAV‐F4/80‐shTLR4 (Figure 9B,D). En face Oil Red O staining of the entire aorta revealed a significant reduction in lesion area in HFD‐fed *ApoE^−/−^* mice treated with shTLR4, compared with HFD‐fed *ApoE^−/−^* mice administered the empty vector (Figure 9E,F). Similarly, HE, Oil Red O and Masson's trichrome staining of aortic root sections revealed that HFD‐fed *ApoE^−/−^* mice treated with shRNA targeting TLR4 exhibited a significantly reduced lesion area in the aortic sinus compared with HFD‐fed *ApoE^−/−^* mice treated with empty vector (Figure 9G,H). Specifically, both necrotic core area and fibrous cap thickness were also markedly decreased in *ApoE^−/−^* mice treated with shRNA targeting TLR4 compared with *ApoE^−/−^* mice treated with empty vector (Figure 9I). Taken together, TLR4/MyD88/NF‐κB/NLRP3 play a critical role in the development of atherosclerotic plaques.

To elucidate the upstream and downstream regulatory relationships among the four key proteins in the TLR4/MyD88/NF‐κB/NLRP3 signalling axis, we conducted a functional rescue experiment in ox‐LDL‐stimulated RAW 264.7 macrophages transduced with AAV‐F4/80‐shTLR4, with or without co‐treatment with the NF‐κB activator berubicin or the NLRP3 activator nigericin. The mRNA and protein expression levels of TLR4 were significantly downregulated in ox‐LDL‐stimulated macrophages following TLR4 silencing mediated by AAV‐F4/80‐shTLR4 (Figure 9J,K). The protein expression of MyD88 was reduced following TLR4 silencing, but remained unchanged upon berubicin treatment (Figure 9L,M). Silencing TLR4 depressed, conversely, berubicin enhanced the expression of pNF‐κB and NLRP3 on ox‐LDL‐treated macrophages (Figure 9L,M). Berubicin partially abolished the inhibition of pNF‐κB and NLRP3 caused by silencing TLR4 (Figure 9L,M). The protein expressions of MyD88 and pNF‐κB were downregulated after TLR4 silencing but remained constant upon berubicin treatment (Figure 9N,O). The knockdown of TLR4 with AAV‐F4/80‐shTLR4 reduced NLRP3 expression in ox‐LDL‐treated macrophages, whereas NLRP3 agonist nigericin enhanced its expression (Figure 9N,O). The downregulation of NLRP3 induced by silencing TLR4 was abolished by nigericin (Figure 9N,O). Collectively, the key signalling pathway TLR4/MyD88/NF‐κB/NLRP3 exhibits well‐defined upstream and downstream regulatory relationships.

### 3.11 Galectin‐3 deficiency or knockdown reduces atherosclerotic lesions in *ApoE* ^−/−^ mice fed by HFD

**FIGURE 10:**
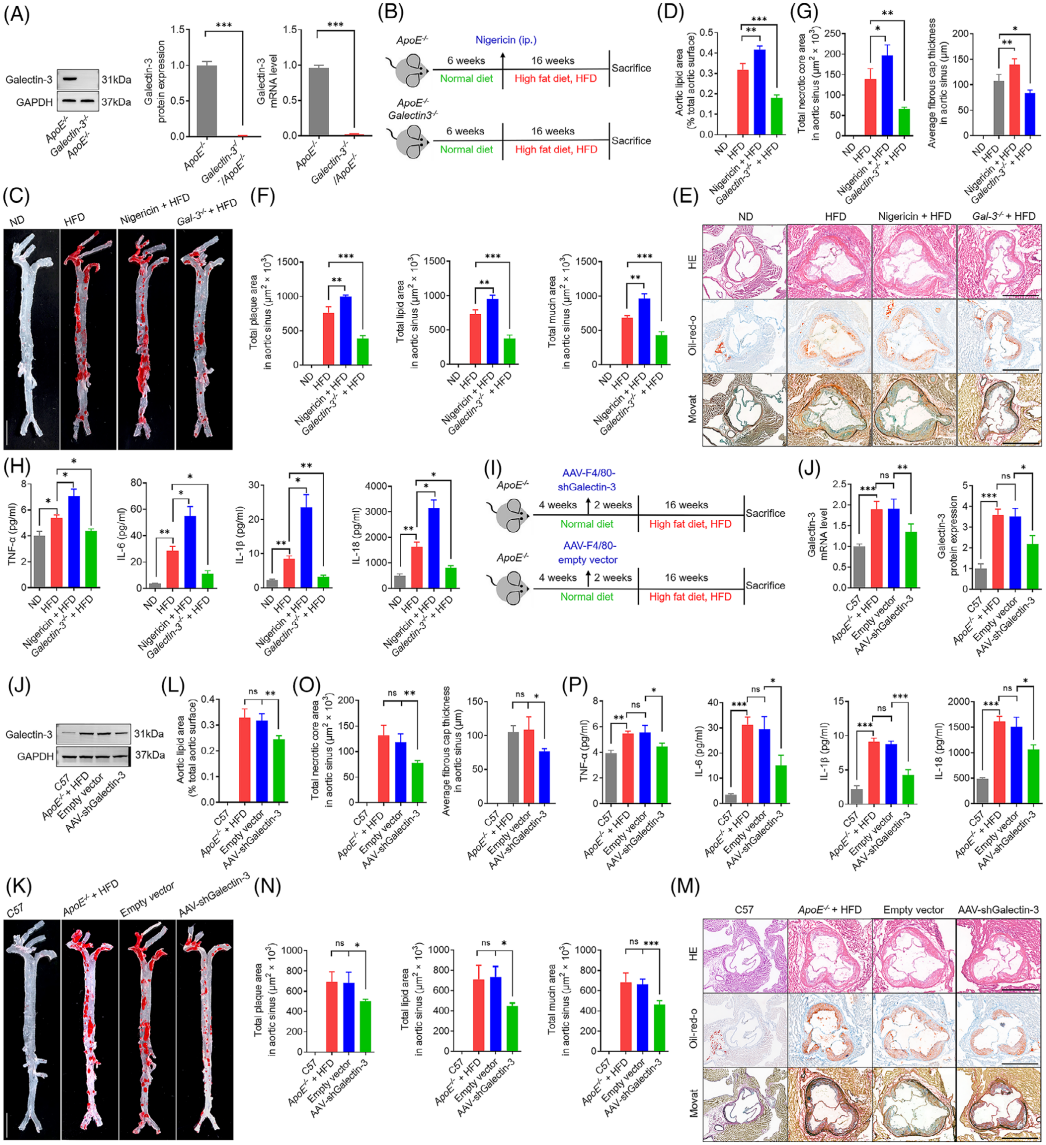
Galectin‐3 genetic deficiency or knockdown reduced and, conversely, NLRP3 agonist nigericin augmented atherosclerotic lesions in HFD‐fed *ApoE^−/−^* mice. (A) The knockout efficacy of galectin‐3 in the aorta is verified by Western blotting and RT‐qPCR. (B) Schematic diagram of animal study design. *Galectin‐3^−/−^/ApoE^−/−^* mice and *ApoE^−/−^* mice are fed an HFD for 16 weeks, and *ApoE^−/−^* mice are intraperitoneally administered with NLRP3 agonist nigericin. (C) Representative images of en face Oil Red O staining in the entire aortas are obtained from *ApoE^−/−^* control mice, HFD‐fed *ApoE^−/−^* mice, HFD‐fed *ApoE^−/−^* mice treated with nigericin, and HFD‐fed *Galectin‐3^−/−^/ApoE^−/−^* mice. Scale bar: 50 mm. (D) En face lesion area is quantified as a percentage of the total area of the aorta. Compared with those in HFD‐fed *ApoE^−/−^* mice, en face lesion areas are significantly smaller in HFD‐fed *Galectin‐3^−/−^/ApoE^−/−^* mice and, conversely, are markedly bigger in HFD‐fed *ApoE^−/−^* mice treated with nigericin. (E) Representative sections of HE, Oil Red O and Movat's staining in the aortic sinuses are acquired from four different groups of mice. Scale bar: 1 mm. (F) Aortic sinus plaque lesion area, lipid lesion area and mucin area are represented as total area in µm^2^. Plaque area, lipid lesion area (red) and mucin area (blue‐green) are much bigger in HFD‐fed *ApoE^−/−^* mice treated with nigericin and, conversely, are relatively smaller in HFD‐fed *Galectin‐3^−/−^/ApoE^−/−^* mice in comparison with HFD‐fed *ApoE^−/−^* mice. (G) The levels of inflammatory cytokines in the aortas are measured from four different groups of mice. (H) Schematic illustration of experimental protocol in *ApoE^−/−^* mice receiving the injection of AAV‐F4/80 shGalectin‐3/empty vector at the age of 4 weeks. After 2 weeks of a normal diet for rest, these mice are treated with HFD for 16 weeks. (I) The knockdown efficacy of shGalectin‐3 in the aorta is confirmed through Western blotting and RT‐qPCR. (J) Representative images of en face Oil Red O staining in the entire aortas are obtained from HFD‐fed *ApoE^−/−^* mice, empty vector‐treated HFD‐fed *ApoE^−/−^* mice and shGalectin‐3‐treated HFD‐fed *ApoE^−/−^* mice. Scale bar: 50 mm. (K) En face lesion area, quantified as a percentage of total area of the aorta, is significantly smaller in shGalectin‐3‐treated HFD‐fed *ApoE^−/−^* mice than in empty vector‐treated HFD‐fed *ApoE^−/−^* mice. (L) Representative sections of HE, Oil Red O and Movat's staining in the aortic sinuses are acquired from three different groups of mice. Scale bar: 1 mm. (M) Aortic sinus plaque lesion area, lipid lesion area (red) and mucin area (blue‐green), represented as total area in µm^2^, are much bigger in shGalctin‐3‐treated HFD‐fed *ApoE^−/−^* mice than in empty vector‐treated HFD‐fed *ApoE^−/−^* mice. (N) The levels of inflammatory cytokines (TNF‐1α, IL‐1β, IL‐18 and IL‐6) in the aortas are measured from three different groups of mice. *n* = 4–8 mice per group. ^*^ *p* ˂.05, ^**^ *p* ˂.01, ^***^p ˂.001 by Student's *t* test. ns: not significant.

According to the activation of galectin‐3 in atherosclerosis, we wanted to determine whether galectin‐3 deficiency inhibits atherosclerotic development. We crossed *Galectin‐3^−/−^* mice with *ApoE^−/−^* mice, that is, galec*tin‐3^−/−^/ApoE^−/−^* mice. Subsequently, we verified the gene deletion efficacy of galectin‐3 in aortic tissue by Western blotting and RT‐qPCR (Figure 10A). The *Galectin‐3^−/−^/ApoE^−/−^* mice were maintained on an HFD for 16 weeks. To further investigate the effect of NLRP3 on atherosclerosis, we administered NLRP3 agonist nigericin to *ApoE^−/−^* mice while continuing to feed HFD for 16 weeks (Figure 10B). Analysis of en face lesion area in the whole aorta indicated that genetic deletion of galectin‐3 significantly reduced, and conversely, NLRP3 agonist treatment increased lesion development in comparison with *ApoE^−/−^* mice fed an HFD (Figure 10C,D). Aortic root sections were stained by Oil Red O, HE and Movat's method. The sinus lesion area was relatively much smaller in *Galectin‐3^−/−^/ApoE^−/−^* mice, and conversely, quite larger in NLRP3 agonist nigericin‐treated *ApoE^−/−^* mice in comparison with *ApoE^−/−^* mice, when mice were fed an HFD for 16 weeks to induce the atherosclerotic model (Figure 10E,F). Similarly, galectin‐3 deficiency was markedly reduced, conversely, an NLRP3 agonist nigericin enlarged necrotic core area and fibrous cap thickness in *ApoE^−/−^* mice fed by HFD (Figure 10G). Consistently, the levels of inflammatory cytokine (TNF‐α, IL‐6, IL‐18 and IL‐1β) were elevated in the aortic tissue of *ApoE^−/−^* mice fed an HFD, which were further exacerbated by NLRP3 agonist nigericin and, conversely, alleviated in *Galectin‐3^−/−^/ApoE^−/−^* mice (Figure 10H).

To highlight the functional drive of macrophage‐derived galectin‐3 during atherosclerotic plaque development in vivo, 4‐week‐old *ApoE^−/−^* mice were injected with AAV‐mediated transfer of shRNA targeting the galectin‐3 gene driven by the macrophage‐specific surface F4/80 antigen (AAV‐F4/80‐shGalectin‐3) or control (AAV‐empty vector) via the tail vein (Figure 10I). These mice were fed an ND for 2 weeks of rest and then fed an HFD for 16 weeks to induce atherosclerosis (Figure 10I). The application of AAV targeted peripheral macrophages to knock down the corresponding protein. The expression of galectin‐3 was measured in aortic tissue after 16 weeks of HFD, and the reduced RNA and protein levels were confirmed by Western blotting and RT‐qPCR, suggesting that AAV‐F4/80‐shGalectin‐3 is highly efficient in silencing galectin‐3 expression in vivo (Figure 10J). Assessment of en face Oil Red O staining of the entire aorta exhibited that the lesion area was diminished in HFD‐fed *ApoE^−/−^* mice treated by shGalectin‐3 compared to HFD‐fed *ApoE^−/−^* mice treated by empty vector (Figure 10K,L). Similarly, HE, Oil Red O and Masson's stainings of aortic root sections revealed that HFD‐fed *ApoE^−/−^* mice treated by shGalectin‐3 had a significantly reduced lesion area in the aortic sinus relative to HFD‐fed *ApoE^−/−^* mice treated by empty vector (Figure 10M,N). Specifically, in vivo silencing of galectin‐3 expression decreased necrotic core area and fibrous cap thickness in the aortic sinus of HFD‐fed *ApoE^−/−^* mice (Figure 10O). In addition, the levels of inflammatory cytokines were markedly inhibited in HFD‐fed *ApoE^−/−^* mice treated by shGalectin‐3 (Figure 10P). Collectively, these results strongly support the notion that galectin‐3 markedly affects inflammatory infiltration and plaque development in HFD‐fed *ApoE^−/−^* mice.

F4/80 has been used widely as a marker for murine macrophages. Theoretically, AAV‐F4/80‐shGalectin‐3 is specifically designed to target RAW 264.7 murine macrophages in order to knock down the corresponding galectin‐3. We utilised macrophage‐specific AAV‐F4/80‐shGalectin‐3 to treat macrophages, smooth muscle cells (SMCs) and endothelial cells (ECs). As shown in Figure S8, the mRNA and protein levels of galectin‐3 were significantly reduced in macrophages treated by AAV‐F4/80‐shGalectin‐3 (Figure S8A,D). Notably, the mRNA and protein levels of galectin‐3 remained consistent in both ECs (Figure S8B,E) and SMCs (Figure S8C,F), regardless of whether AAV‐F4/80‐shGalectin‐3 was administered or not. The aforementioned results suggest that the application of AAV‐F4/80‐shGalectin‐3 specifically targets macrophages, with no appreciable transduction of SMCs and ECs. Immunofluorescence detection of galectin‐3‐positive macrophages, using a F4/80 and galectin‐3 antibody, showed substantially reduced staining in lesions at the aortic roots of HFD‐fed *ApoE^−/−^* mice treated by shGalectin‐3 compared to those of HFD‐fed *ApoE^−/−^* mice treated by empty vector or not (Figure S8G).

## 4 DISCUSSION

PANoptosis is a unique inflammation‐regulated cellular death that is driven and modulated by multiprotein PANoptosome complex formation, which exhibits critical characteristics of necroptosis, pyroptosis and apoptosis that cannot be fully defined by any one of these three major pathways alone. PANoptosis has involved a wide range of human diseases comprising microbial infections, cancer, autoinflammatory diseases, neurodegenerative diseases and metabolic conditions. Bacterial or viral infections of macrophages initiate PANoptosis and the formation of a multiprotein PANoptosome complex, including pyroptotic, apoptotic and necroptotic components to regulate the PANoptosis.^25^ Vrial infections induced macrophage pyroptosis, apoptosis and necroptosis together, revealing extensive crosstalk and coregulation among these three programmed cellular death pathways, proposing the concept of PANoptosis.^26^ Bacterial pathogens activated pyroptosis, apoptosis and necroptosis in macrophages via the RIPK1‐mediated PANoptosis.^27^ Pyroptosis, apoptosis and necroptosis (PANoptosis) were reduced in colorectal cancer, and the modulation of PANoptosis suppressed tumorigenesis.^28^, ^29^ Inflammatory cellular death leads to the release of multiple intracellular cytokines, which positively accelerate the inflammatory process and associated response. Our experimental data revealed that human and animal atherosclerotic lesions were accompanied by the substantial release of proinflammatory mediators, the upregulation of key proteins related to the pyroptotic, apoptotic and necroptotic signalling pathways, and the typical ultrastructural features of PANoptosis‐like cell death using TEM. We further verified the colocalisation of macrophage marker CD68 or F4/80 and the key proteins (GSDMD/RIPK3/CASP3), suggesting that pyroptosis, apoptosis and necroptosis existed in macrophages. These are in line with our in vitro cell data that ox‐LDL induced the changes in ultrastructural morphology, the upregulation of key proteins and the extracellular release of proinflammatory mediators, which indicated the simultaneous occurrences of necroptosis, pyroptosis and apoptosis in macrophages.

Necroptosis, controlled by RIPK1, RIPK3 and MLKL, in plaque macrophages, contributes considerably to the release of proinflammatory mediators and the expansion of the necrotic core. We demonstrated the presence of necroptotic cells and the elevated levels of RIPK3 and pMLKL in advanced atherosclerotic lesions. In vitro study found the treatment of macrophages with ox‐LDL resulted in necroptotic cellular death, as manifested by the upregulation of RIPK3, the promotion of MLKL phosphorylation, the rupture of the plasma membrane and translucent electron‐light cytoplasm under a TEM. These are consistent with a prior study demonstrating that the expression levels of necroptotic genes RIPK3 and MLKL are markedly elevated in carotid atherosclerotic plaques, and ox‐LDL significantly induces the phosphorylation of RIPK3 and MLKL in macrophages.^30^ RIPK1 was highly expressed in macrophage foam cells within early‐stage atherosclerotic aortas of both humans and mice.^31^ The expression level of RIPK3 was significantly augmented in the atherosclerotic aortas of *ApoE^−/−^* mice following 12–20 weeks of HDF feeding.^32^ Therapeutic targeting of macrophage necroptosis may offer beneficial effects on the inhibition of atherosclerosis. The necroptosis inhibitor necrostatin‐1 (Nec‐1) diminished the development of atherosclerotic lesions in 10‐week HFD‐fed *ApoE^−/−^* mice.^30^ Therapeutic knockdown of RIPK1 resulted in a 47% reduction in the area of early‐stage atherosclerosis in *ApoE^−/−^* mice fed by a western diet for 8 weeks.^31^ RIPK3 gene deficiency reduced the area and thickness of the atherosclerotic plaque, the release of proinflammatory factors within the plaques, and the mortality of *ApoE^−/−^* mice fed by an HFD.^32^ Similarly, genetic deletion of RIPK3 was shown to lead to a 50% decrease in necrotic macrophages and a 60% reduction in necrotic core area in *ApoE^−/−^* mice on the HFD for 16 weeks.^33^ MLKL genetic knockdown reduced the size of the necrotic core by over 50% in *ApoE^−/−^* mice fed by an HFD for 16 weeks.^34^

Pyroptosis represents a novel caspase‐1‐dependent inflammatory modality of cell death. Activated caspase‐1 cleaves GSDMD, facilitating GSDMD‐N to penetrate the cellular membrane and promoting the release of intracellular molecules. Consistent with prior studies, we found the presence of pyroptotic cells and the elevated expression of NLRP3 and GSDMD‐N, and the colocalisation of GSDMD/GSDMD‐N with a macrophage marker in atherosclerotic lesions from human and mice. The pyroptosis component caspase‐1, ASC, NLRP3, GSDMD, IL‐18 and IL‐1β were abundantly expressed in carotid artery atherosclerotic plaques but not in healthy peripheral arteries; moreover, their expression levels were markedly elevated in unstable relative to stable plaques.^35^ The NLRP3 inflammasome was predominantly localised to the cytoplasm of macrophage‐derived foam cells within the atherosclerotic core of carotid plaques.^35^ The NLRP3 expression was demonstrated to be significantly elevated in the ascending aorta of patients with severe coronary artery stenosis when compared to arterial tissue without atherosclerosis.^36^ NLRP3 inflammasome activation is involved in the inflammatory damage in the kidney and lung.^37^, ^38^ In this study, we demonstrated that ox‐LDL activated pyroptosis in THP‐1 macrophages manifested by plasma membrane pore formation, consequent release of cytosolic contents and the robust inflammatory responses. Consistent with our findings, Ox‐LDL treatment elevated the expression of GSDMD and caspase‐1, increased the IL‐1β and IL‐18 release, and destroyed the cell membrane integrity, thereby inducing macrophage pyroptosis.^39^, ^40^ Therefore, therapeutic targeting of inflammatory macrophage pyroptosis is proposed to mitigate vascular inflammation and atherosclerosis. Caspase‐1 gene deficiency significantly diminished the atherosclerotic plaque area, the number of macrophage cells within the plaques, and the serum release of various proinflammatory cytokines in *ApoE^−/−^* mice fed by an HFD.^41^, ^42^ Caspase‐1 inhibitor VX765 counteracted NLRP3 inflammasome assembly, mitigated pyroptotic cell death in macrophages, and retarded necrotic core formation in HFD‐fed *ApoE^−/−^* mice.^43^ Lentivirus‐mediated NLRP3 gene silencing reduced inflammatory cytokines, mitigated atherosclerotic plaque progression, and decreased plaque vulnerability in *ApoE^−/−^* mice fed with HFD for 8 weeks.^44^ NLRP3 inhibitor MCC950 suppressed the activation of NLRP3/ASC/caspase‐1/GSDMD‐N signalling axis, mitigated macrophage pyroptosis and reduced the plaque area in *ApoE^−/−^* mice fed with HFD for 12 weeks.^45^

Apoptosis is a widely acknowledged phenomenon in atherosclerotic lesions. Our study assessed the expression and activation of two apoptosis proteases, caspase‐3 and caspase‐8, in association with apoptotic cells within advanced atherosclerotic lesions. In the apoptotic cascade, the activation and cleavage of caspase‐3 and caspase‐8 are established as crucial mediators. Apoptosis is associated with mitochondrial dynamic disturbance, endoplasmic reticulum stress, oxidative stress and cell cycle dysregulation.^46^, ^47^ This study demonstrated the strong expression of the caspase‐3 and caspase‐8 precursors, along with their cleavage fragment complements, and revealed immunofluorescence colocalisation of caspase‐3 with macrophage marker‐positive regions in advanced atherosclerotic lesions of human and mice. Ultrastructural analysis by TEM confirmed the occurrence of apoptosis with the features of cytoplasmic shrinkage and nuclear condensation in advanced atherosclerotic lesions. Positive staining for caspase‐3 was markedly elevated in coronary unstable atherosclerotic plaques relative to stable atherosclerotic plaques.^48^ Caspase‐8 is an essential protease that classically initiates the extrinsic apoptotic pathway upon the activation of cell surface death receptors.^49^ Caspase‐8 regulates the interplay among apoptosis, necroptosis and pyroptosis, therefore determining the mode of cellular death in response to cellular death signals.^50^ In our study, ox‐LDL‐treated macrophages exhibited early morphological alteration characteristics consistent with the nuclear changes associated with apoptosis. Macrophage apoptosis following ox‐LDL treatment contributes to the development of vulnerable plaques and compromises the integrity of the fibrous cap.^51^ Apoptotic macrophages were found to be present at the site of culprit coronary plaque rupture in patients with sudden cardiac death.^52^ Efferocytosis is defined as the normal physiological process of phagocytic clearance of apoptotic cells. Phagocytosis of apoptotic cells was impaired in advanced atherosclerotic lesions,^53^ macrophage apoptosis, coupled with impaired efferocytosis, led to the accumulation of nonengulfed apoptotic cells, proinflammatory response and necrotic core formation.^54^ Protecting macrophage cells from apoptosis is advantageous for inhibiting advanced plaque progression.^55^ Caspase‐3 inhibitor led to a significant reduction in macrophage apoptosis within atherosclerotic lesions.^56^

Macrophages are critical in atherosclerotic plaque formation. Compared with the proximal artery (PA), the atherosclerotic core (AC) contained a higher proportion of macrophages. Single‐cell RNA sequencing on *ApoE^−/−^* mice versus HFD‐fed *ApoE^−/−^* mice aortas revealed that the proportion of monocytes/macrophages was increased following HFD treatment from 17.42% to 56.14%.^57^ Macrophages serve as the main source of galectin‐3 expression, indicating that galectin‐3 is critical for inflammation and atherosclerosis. Immunofluorescence colocalisation of galectin‐3 with macrophage marker in atherosclerotic plaques further substantiates our findings. In vivo, we showed that galectin‐3 was obviously upregulated in advanced atherosclerotic lesions of humans and mice. Galectin‐3 is mainly expressed in macrophages. In vitro, we further found that ox‐LDL promoted galectin‐3 expression in macrophages, and galectin‐3 contributed to NLRP3 inflammasome activation. Previous studies have linked atherosclerosis with increased levels of galectin‐3. The expression level of galectin‐3 was statistically higher in atherosclerosis lesions obtained from carotid endarterectomies and lower limb amputation compared with that in umbilical arteries; moreover, galectin‐3 was predominantly localised to macrophage‐derived foam cells within human atherosclerotic lesions.^58^ Galectin‐3 expression is markedly elevated in the vulnerable sites of human atherosclerotic plaques and in murine atherosclerotic plaques, compared with their invulnerable sites and wild‐type controls.^59^ Galectin‐3 immunostaining colocalised with macrophages, and its mRNA expression level was significantly correlated with the macrophage marker CD68 expression.^59^ Galectin‐3 and NLRP3 were highly upregulated in human carotid atherosclerotic plaques and in atherosclerotic aortas from HFD‐fed *ApoE^−/−^* mice; moreover, galectin‐3 bound to NLRP3 to accelerate inflammation activation and atherosclerotic progression.^60^ Inhibition of galectin‐3 with a specific galectin‐3 inhibitor TD139, effectively suppressed NLRP3 expression, attenuated inflammation and oxidative stress, and reduced cytokine levels.^61^, ^62^ In vitro, we found that galectin‐3 induced macrophage apoptosis, pyroptosis through its interaction with NLRP3. In vivo, we further noted that galectin‐3 genetic deficiency or knockdown suppressed PANoptosis‐related inflammation and attenuated atherosclerosis in HFD‐fed *ApoE^−/−^* mice. The attenuated atherogenesis observed in galectin‐3‐deficient or knockdown models is closely relevant to its function as a critical regulator of macrophage cell death. Conversely, NLRP3 activator induced macrophage pyroptosis, apoptosis and necroptosis, and was associated with significant development and progression of atherosclerotic lesions in *ApoE^−/−^* mice fed by HFD. These findings are consistent with our in vitro cell data and line with prior studies indicating that galectin‐3 was upregulated in ox‐LDL‐induced macrophages.^63^ We found that ox‐LDL stimulation induced inflammatory cytokine release and PANoptosis‐like death in macrophages. Knockdown of galectin‐3 alleviated the detrimental impact of ox‐LDL on macrophages, whereas NLRP3 activator enhanced these effects. Galectin‐3 modulated the endocytosis of ox‐LDL, resulting in the intracellular accumulation of cholesteryl esters, and contributed to foam cell formation.^64^

Galectin‐3 protein, encoded by the Lgals3 gene, is localised to the plasma membrane, cytoplasm, nucleus and the extracellular space.^65^ Galectin‐3 is principally secreted by macrophages and activates these cells in an autocrine or paracrine fashion.^66^ Activated macrophages subsequently secrete galectin‐3, causing a positive feedback amplification effect. Galectin‐3 is critical for cellular proliferation, apoptosis, migration, pyroptosis and inflammation.^67^, ^68^ Our study found that galectin‐3 was upregulated in response to HFD feeding or ox‐LDL stimulation, and that galectin‐3 activated NLRP3 inflammasome directly or through the intermediate protein, including TLR4, MyD88 and NF‐κB. Galectin‐3 has been shown to function as a ligand for TLR4 and bind to TLR4 on the cell membrane.^69^ TLR4 upregulated the expression of the adapter molecule MyD88 and the phosphorylation of NF‐κB, subsequently activating NLRP3 inflammasome.^70^ The inhibition of galectin‐3 specifically reduced the expression of TLR4, NF‐κB, pNF‐κB, NLRP3 and GSDMD in microglia treated with LPS.^71^ Our in vitro study demonstrated that galectin‐3 knockdown inhibited the expression of TLR4, MyD88, and the phosphorylation of NF‐κB, which alleviated the apoptosis, pyroptosis and necroptosis in ox‐LDL‐treated macrophages. Functional recovery experiments revealed that TLR4 genetic knockdown negatively signalled to the MyD88/NF‐κB/NLRP3 axis, which was effectively reversed by either the NF‐κB activator berubicin or the NLRP3 activator nigericin. Moreover, our in vivo animal study indicated that galectin‐3 gene deficiency inhibited the TLR4/MyD88/NF‐κB signalling pathway, resulting in a reduction in atherosclerosis. On the other hand, NLRP3 agonist nigericin did not change the expression of the upstream TLR4, MyD88 and NF‐κB. We further found that in vivo TLR4 genetic knockdown effectively alleviated the development of atherosclerosis via the downregulation of MyD88/pNF‐κB/NLRP3 signals in HFD‐fed *ApoE^−/−^* mice. The TLR4/MyD88/NF‐κB/NLRP3 signalling axis plays a critical role in the pathogenesis and progression of atherosclerosis.

### 4.1 Limitation

First, galectin‐3 is constitutively expressed by multiple cell types, containing endothelial cells, smooth muscle cells, neutrophils, NK‐T cells, T cells, B cells and fibroblasts, all of which are present in atherosclerotic lesions and contribute to atherosclerosis. Thus, a potential impact of galectin‐3 on these cell types during atherosclerosis cannot be completely ruled out. Second, the co‐existence of necroptosis, pyroptosis and apoptosis allows for extensive crosstalk and intricate interaction among several cellular death pathways, which cannot be seriously disrupted by the inhibitor of an individual pathway alone. The colocalisation of GSDMD, caspase‐3 and RIPK3, as revealed by double or triple immunofluorescence staining, is insufficient to conclusively establish the occurrence of PANoptosis in macrophages. The present study represents only an initial step toward understanding PANoptosis‐like cell death in atherosclerosis. Third, PANoptosis represents a highly coordinated and cooperatively regulated form of programmed cell death that is mediated by a cytoplasmic multiprotein complex named the PANoptosome in response to ox‐LDL and cholesterol crystals. This work did not elucidate mechanistic insights into the critical component and assembly of the PANoptosome complex.

## 5 CONCLUSIONS

Collective activation of necroptosis, pyroptosis and apoptosis occurred in atherosclerotic macrophages. Galectin‐3 expression was significantly upregulated and originated mainly from macrophages during atherosclerosis. Galectin‐3 deficiency suppressed pyroptosis, apoptosis and necroptosis in macrophages and migrated the progression of inflammation and atherosclerosis. Mechanistically, galectin‐3 activates TLR4/MyD88/NF‐κB/NLRP3 signalling axis and contributes to PANoptosis‐like macrophage death. Targeting galectin‐3/TLR4/MyD88/NF‐κB/NLRP3 signalling axis represents a promising therapeutic approach for the management of atherosclerosis.

## AUTHOR CONTRIBUTIONS

**Zihui Yuan** performed research, contributed new reagents or analytic tools, and analysed data; **Haitao Li** performed research and analysed data; **Hongyi Huang** analysed data; **Yiqing Li** designed research and analysed data; **Jian Wang** designed research, analysed data and wrote the paper. All authors have read and approved the submitted manuscript.

## CONFLICT OF INTEREST STATEMENT

The authors declare no conflicts of interest.

## ETHICS STATEMENT

All the ethical considerations and experimental programmes have been approved by the Ethics Committee of Union Hospital and Huazhong University of Science and Technology (IEC‐485, 6 March 2023). The work described has been carried out in accordance with the Code of Ethics of the World Medical Association (Declaration of Helsinki) for experiments involving humans. All animal experiments complied with the ARRIVE guideline (Animal Research: Reporting of In Vivo Experiments) and were carried out in accordance with the National Institutes of Health guide for the care and use of laboratory animals (NIH publications No. 8023, revised 1978).

## Supporting information




**Figure S1** Proteomic and transcriptome characterisation of the aortas of *ApoE^−/−^* mice versus HFD‐fed *ApoE^−/−^* mice. (A) Pearson correlation coefficient was indicative of significant variation in the proteomic levels among the biological replicates. (B) Principal component analysis revealed a significant separation within the proteomic data between *ApoE^−/−^* mice and HFD‐fed *ApoE^−/−^* mice. Each point represents an individual sample forming two distinct clusters. (C) Heatmap displays the expression patterns of the DEPs. Colour intensity represents the expression levels, with blue indicating lower expression and orange indicating higher expression. Points above the horizontal line indicate the DEPs (*p* < .05) with a Log2Fold change ≥1 or less than −1. (D) Compared to *ApoE^−/−^* mice, the gene set upregulated in HFD‐fed *ApoE^−/−^* mice was enriched in the biological processes related to regulation of cell killing, leukocyte chemotaxis, leukocyte migration, leukocyte migration and intrinsic apoptotic signalling. (E) Pearson correlation coefficient approached 1.0, indicating that the data quality was excellent. (F) The score plots of principal component analysis (PCA) show significant differences in transcript abundance of genes between normal and atherosclerosis, despite several common features. (G) The heatmap displays the 225 upregulated DEGs between normal and atherosclerosis in GSE83112.

**Figure S2** Cell clusters and macrophage subtypes are characterised by single‐cell transcriptome analysis of human carotid endarterectomy samples. (A) A heatmap of the top 10 feature genes created for each cell cluster. The colour scale depicts the expression levels of each gene: pink: low, yellow: high. (B) Violin plots of signature genes in different cell types confirm cluster identities. (C) Expression of the top 10 marker genes for the macrophage subcluster is visualised by feature plots of the tSNE. (D) Violin plot of 10 macrophage marker genes constructed for each cell cluster. (E) A dot plot of 10 macrophage canonical marker genes generated for each cell cluster. The dot size represents the percentage of cells expressing the indicated gene. The dot colour scale represents the standardised gene expression level. (F) Heatmap of the top 10 feature genes are generated in each macrophage subtype in comparison with the other three macrophage subtypes. (G) Violin plots of signature genes in different macrophage subtypes confirmed subtype identities.

**Figure S3** Pearson correlation coefficients (PCCs) demonstrating the colocalisation of galectin‐3, NLRP3 and CD68(F4/80) in human and mouse atherosclerotic lesions. (A) Human atherosclerotic lesions showing greater colocalisation of galectin‐3/NLRP3, galectin‐3/CD68 and NLRP3/CD68 compared with normal arterial tissue. (B) Atherosclerotic mouse aortas exhibiting significantly increased colocalisation of galectin‐3/NLRP3, galectin‐3/CD68 and NLRP3/CD68 compared with the normal mouse aorta. Data are derived from three to five independent experiments. ^*^ *p* ˂.05, ^**^ *p* ˂.01, ^***^p ˂.001 by Student's *t* test. ns: not significant.

**Figure S4** Pearson correlation coefficients (PCCs) indicating the colocalisation of caspase‐3, GSDMD and RIPK3 in CD68‐positive macrophages within human atherosclerotic lesions. (A) Human atherosclerotic lesions exhibiting significantly greater colocalisation of caspase‐3/GSDMD, caspase‐3/RIPK3 and GSDMD/RIPK3 compared with normal arterial tissue. (B) The colocalisation of caspase‐3/CD68, GSDMD/CD68 and RIPK3/CD68 are markedly enlarged in human atherosclerotic lesions relative to normal arterial tissue. Data are derived from three to five independent experiments. ^*^ *p* ˂.05, ^**^ *p* ˂.01, ^***^p ˂.001 by Student's *t* test. ns: not significant.

**Figure S5** Ox‐LDL elevated the activity of caspase‐3, caused the nuclear translocation of NF‐κB, and a combination of pyroptosis, apoptosis and necroptosis inhibitors decreased the ox‐LDL‐induced PI‐positive cell death. (A) Ox‐LDL treatment enhanced the activity of caspase‐3, which is mostly recovered by genetic galectin‐3 knockdown. (B) Ox‐LDL treatment initiated the nuclear translocation of NF‐κB, which is markedly reversed by genetic galectin‐3 knockdown. Scale bar: 25 µm. (C) Flow cytometry with PI/Hoechst staining for macrophages conducted with ox‐LDL, along with single, double or triple combinations of Z‐VAD, DSF and Nec‐1. (D) Percentage of PI‐positive cells is calculated in macrophages with ox‐LDL, along with single, double or triple combinations of Z‐VAD, DSF and Nec‐1. Data are derived from three to five independent experiments. ^*^ *p* ˂.05, ^**^ *p* ˂.01, ^***^p ˂.001 by Student's *t* test. ns: not significant.

**Figure S6** Galectin‐3 inhibitor TD139 repressed the activation of pyroptosis, apoptosis and necroptosis in vitro. (A) Representative Western blots and relative quantitative analysis of galectin‐3 in ox‐LDL‐treated macrophages, added by DMSO or DMSO plus TD139. (B) Representative Western blots and relative quantitative analysis of NLRP3, GSDMD and GSDMD‐N in ox‐LDL‐treated macrophages, added by DMSO or DMSO plus TD139. (C) Representative Western blots and relative quantitative analysis of caspase‐3, cleaved caspase‐3, caspase‐8 and cleaved caspase‐8 in ox‐LDL‐treated macrophages, added by DMSO or DMSO plus TD139. (D) Representative Western blots and relative quantitative analysis of RIPK3, MLKL and phospho‐MLKL in ox‐LDL‐treated macrophages, added by DMSO or DMSO plus TD139. Data are derived from three to five independent experiments. ^*^ *p* ˂.05, ^**^ *p* ˂.01, ^***^p ˂.001 by Student's *t* test. ns: not significant.

**Figure S7** Pearson correlation coefficients (PCCs) revealed the colocalisation of caspase‐3, GSDMD and RIPK3 in CD68‐positive macrophages within murine atherosclerotic lesions. (A) The colocalisation of caspase‐3/GSDMD, caspase‐3/RIPK3 and GSDMD/RIPK3 was evident in the atherosclerotic aorta of HFD‐fed *ApoE^−/−^* mice relative to the normal aorta of *ApoE^−/−^* mice. (B) The colocalisation of caspase‐3 with F4/80, GSDMD with F4/80 and RIPK3 with F4/80 were significantly enhanced in the atherosclerotic aorta of HFD‐fed *ApoE^−/−^* mice compared with the normal aorta of *ApoE^−/−^* mice. Data are derived from three to five independent experiments. ^*^ *p* ˂.05, ^**^ *p* ˂.01, ^***^p ˂.001 by Student's *t* test. ns: not significant.

**Figure S8** AAV‐F4/80‐shGalectin‐3 specifically knocked down the corresponding protein in macrophages, while not affect SMCs and ECs in vivo. (A–C) In RAW 264.7 macrophages treated by AAV‐F4/80‐shGalectin‐3, we found a reduction in the protein levels of galectin‐3. In SMCs and ECs, after AAV‐F4/80‐shGalectin‐3, the protein expression of galectin‐3 is unchanged. (D–F) AAV‐F4/80‐shGalectin‐3 effectively and specifically downregulates the mRNA level of galectin‐3 specifically in macrophages, without altering its mRNA levels in ECs or SMCs. (G) Double immunofluorescence staining for galectin‐3 (red), F4/80 (green) and DAPI (blue) in the aortic root of HFD‐fed *ApoE^−/−^* mice demonstrates the location and distribution of galectin‐3‐positive macrophages as indicated by the colocalisation of galectin‐3 and F4/80 (a macrophage marker). Data are derived from three to five independent experiments. ^*^ *p* ˂.05, ^**^ *p* ˂.01, ^***^p ˂.001 by Student's *t* test. ns: not significant.

## Data Availability

The datasets used and/or analysed during the current study are available from the corresponding author upon reasonable request.
