# Supplementary figures and images for: Macrophage‐derived galectin‐3 contributes to pyroptosis, apoptosis and necroptosis through TLR4/MyD88/NF‐κB/NLRP3 during atherosclerosis
Source: Clin Transl Med. 2026 Mar 8;16(3):e70637. doi: 10.1002/ctm2.70637 (PMC12967500; doi:10.1002/ctm2.70637)

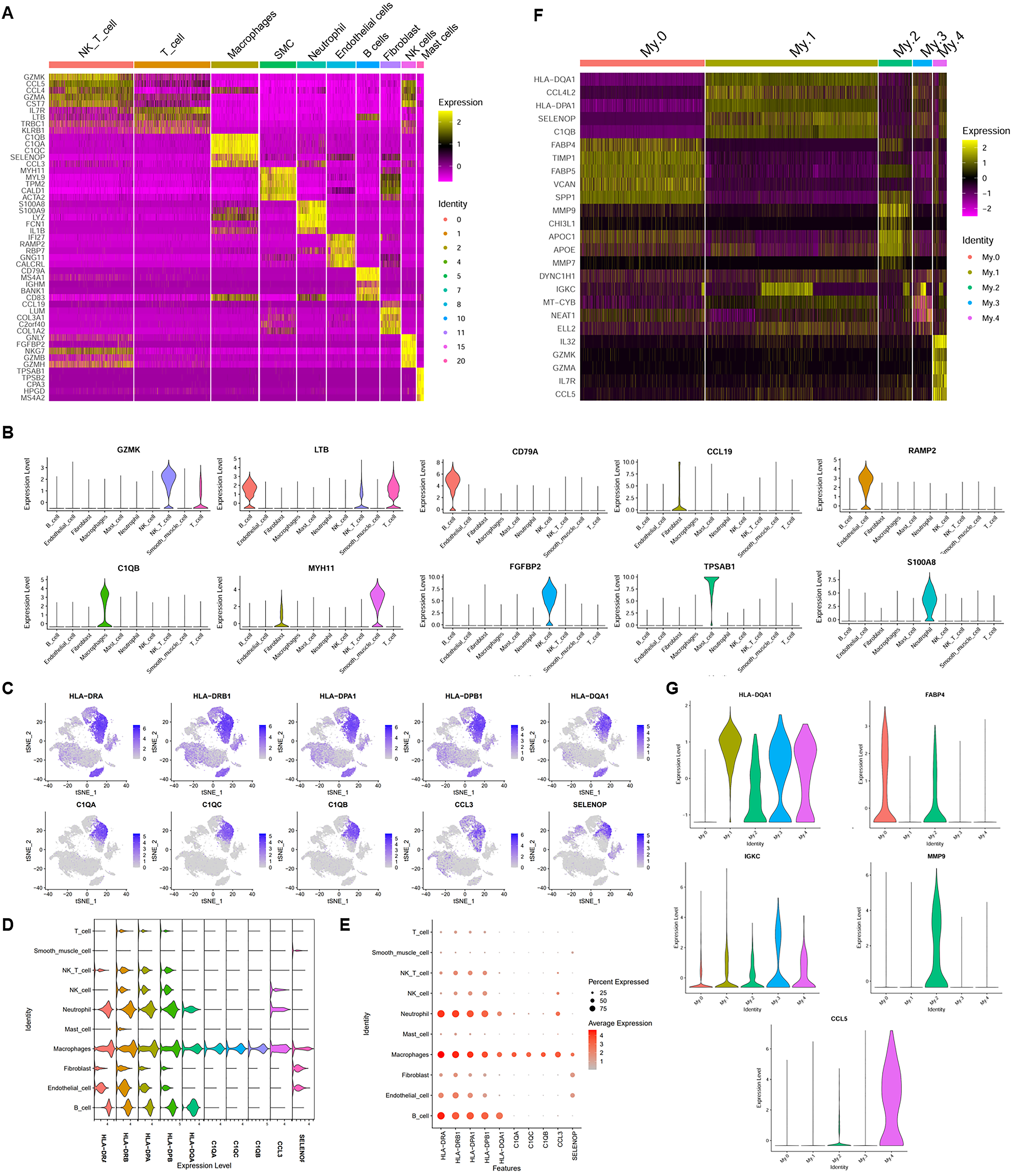

Supplement: Supplementary file 2 — Figure S2 Cell clusters and macrophage subtypes are characterised by single‐cell transcriptome analysis of human carotid endarterectomy samples. (A) A heatmap of the top 10 feature genes created for each cell cluster. The colour scale depicts the expression levels of each gene: pink: low, yellow: high. (B) Violin plots of signature genes in different cell types confirm cluster identities. (C) Expression of the top 10 marker genes for the macrophage subcluster is visualised by feature plots of the tSNE. (D) Violin plot of 10 macrophage marker genes constructed for each cell cluster. (E) A dot plot of 10 macrophage canonical marker genes generated for each cell cluster. The dot size represents the percentage of cells expressing the indicated gene. The dot colour scale represents the standardised gene expression level. (F) Heatmap of the top 10 feature genes are generated in each macrophage subtype in comparison with the other three macrophage subtypes. (G) Violin plots of signature genes in different macrophage subtypes confirmed subtype identities. [file CTM2-16-e70637-s006.png]

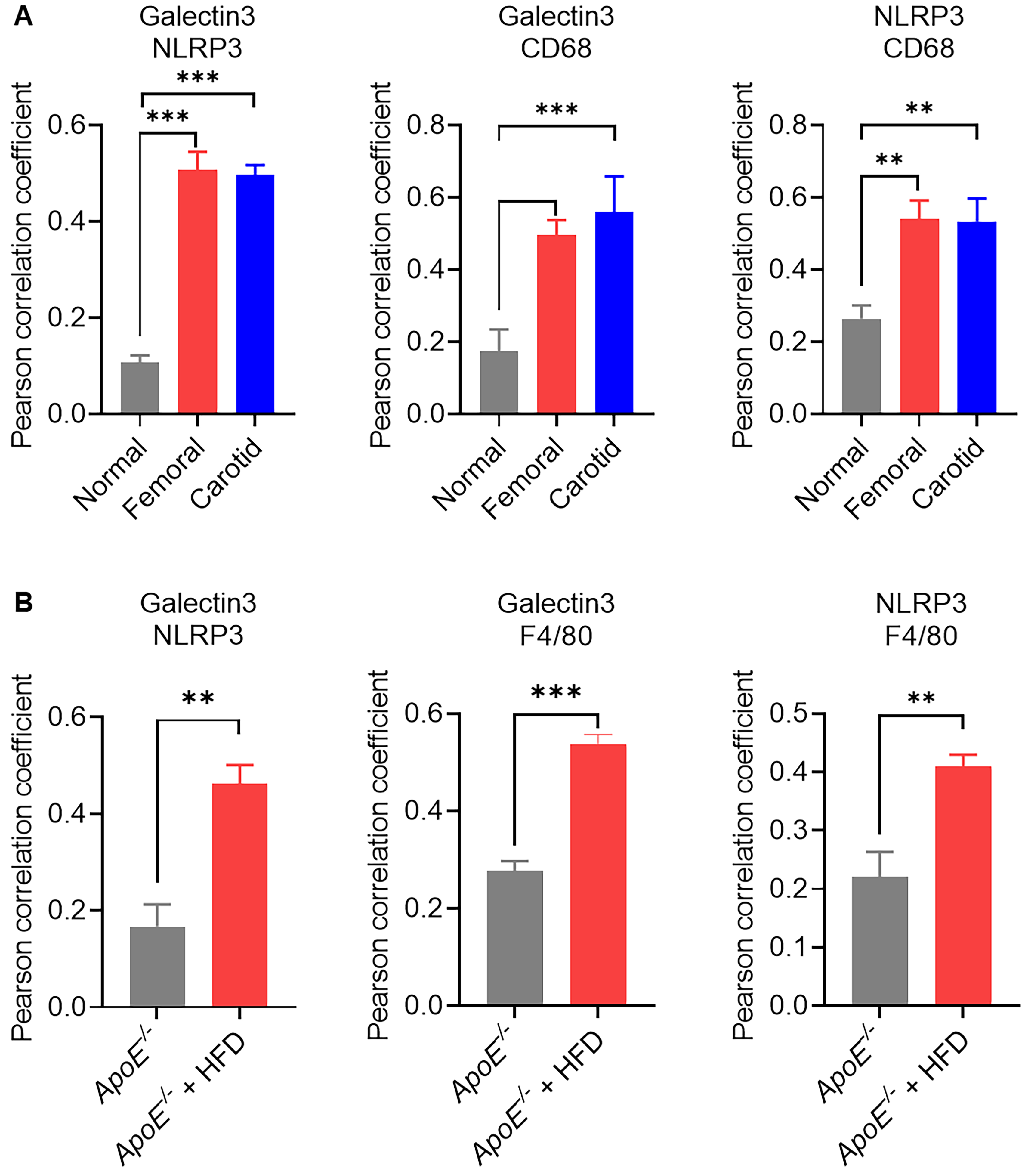

Supplement: Supplementary file 3 — Figure S3 Pearson correlation coefficients (PCCs) demonstrating the colocalisation of galectin‐3, NLRP3 and CD68(F4/80) in human and mouse atherosclerotic lesions. (A) Human atherosclerotic lesions showing greater colocalisation of galectin‐3/NLRP3, galectin‐3/CD68 and NLRP3/CD68 compared with normal arterial tissue. (B) Atherosclerotic mouse aortas exhibiting significantly increased colocalisation of galectin‐3/NLRP3, galectin‐3/CD68 and NLRP3/CD68 compared with the normal mouse aorta. Data are derived from three to five independent experiments. * p ˂.05, ** p ˂.01, ***p ˂.001 by Student's t test. ns: not significant. [file CTM2-16-e70637-s008.png]

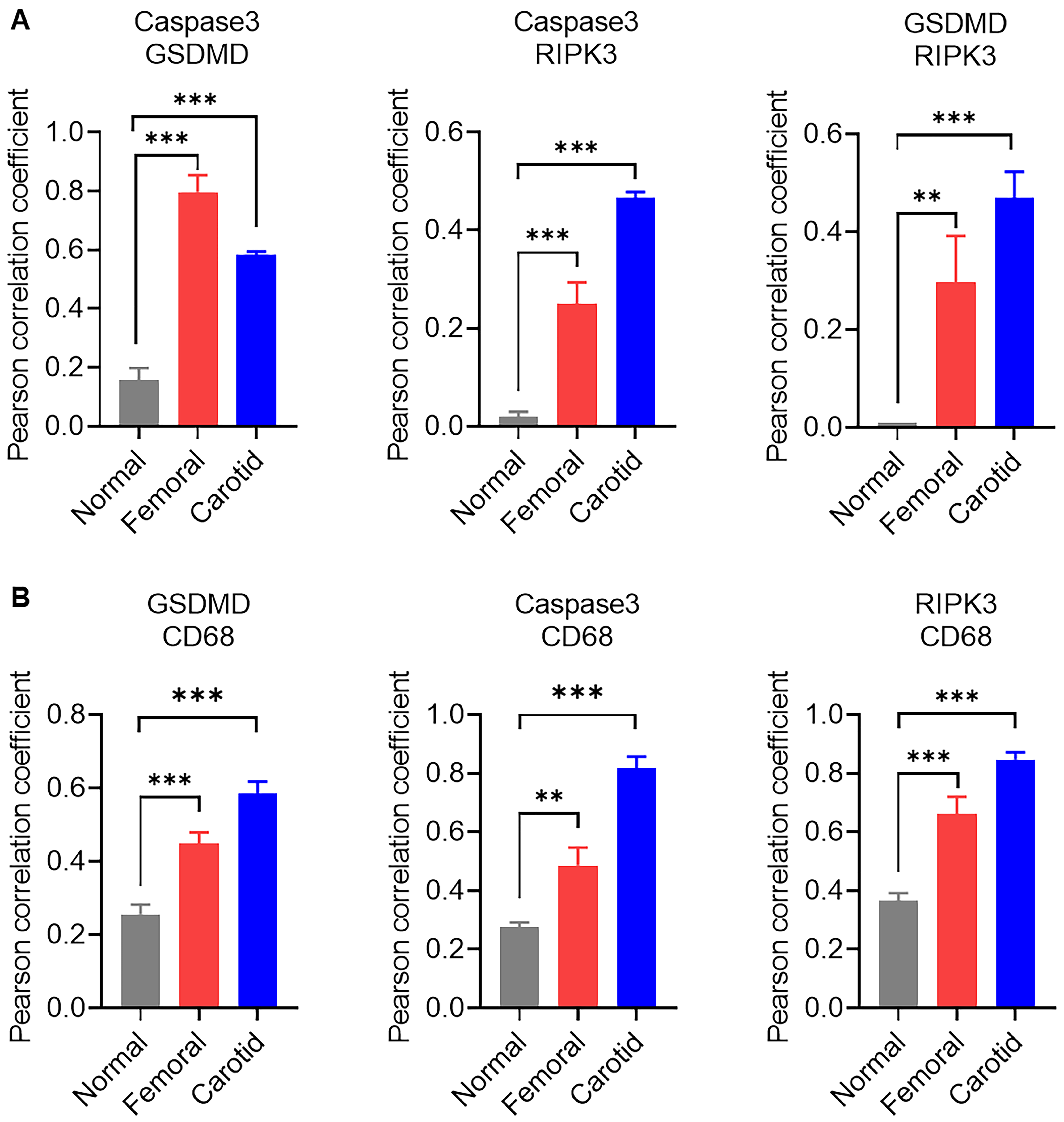

Supplement: Supplementary file 4 — Figure S4 Pearson correlation coefficients (PCCs) indicating the colocalisation of caspase‐3, GSDMD and RIPK3 in CD68‐positive macrophages within human atherosclerotic lesions. (A) Human atherosclerotic lesions exhibiting significantly greater colocalisation of caspase‐3/GSDMD, caspase‐3/RIPK3 and GSDMD/RIPK3 compared with normal arterial tissue. (B) The colocalisation of caspase‐3/CD68, GSDMD/CD68 and RIPK3/CD68 are markedly enlarged in human atherosclerotic lesions relative to normal arterial tissue. Data are derived from three to five independent experiments. * p ˂.05, ** p ˂.01, ***p ˂.001 by Student's t test. ns: not significant. [file CTM2-16-e70637-s007.png]

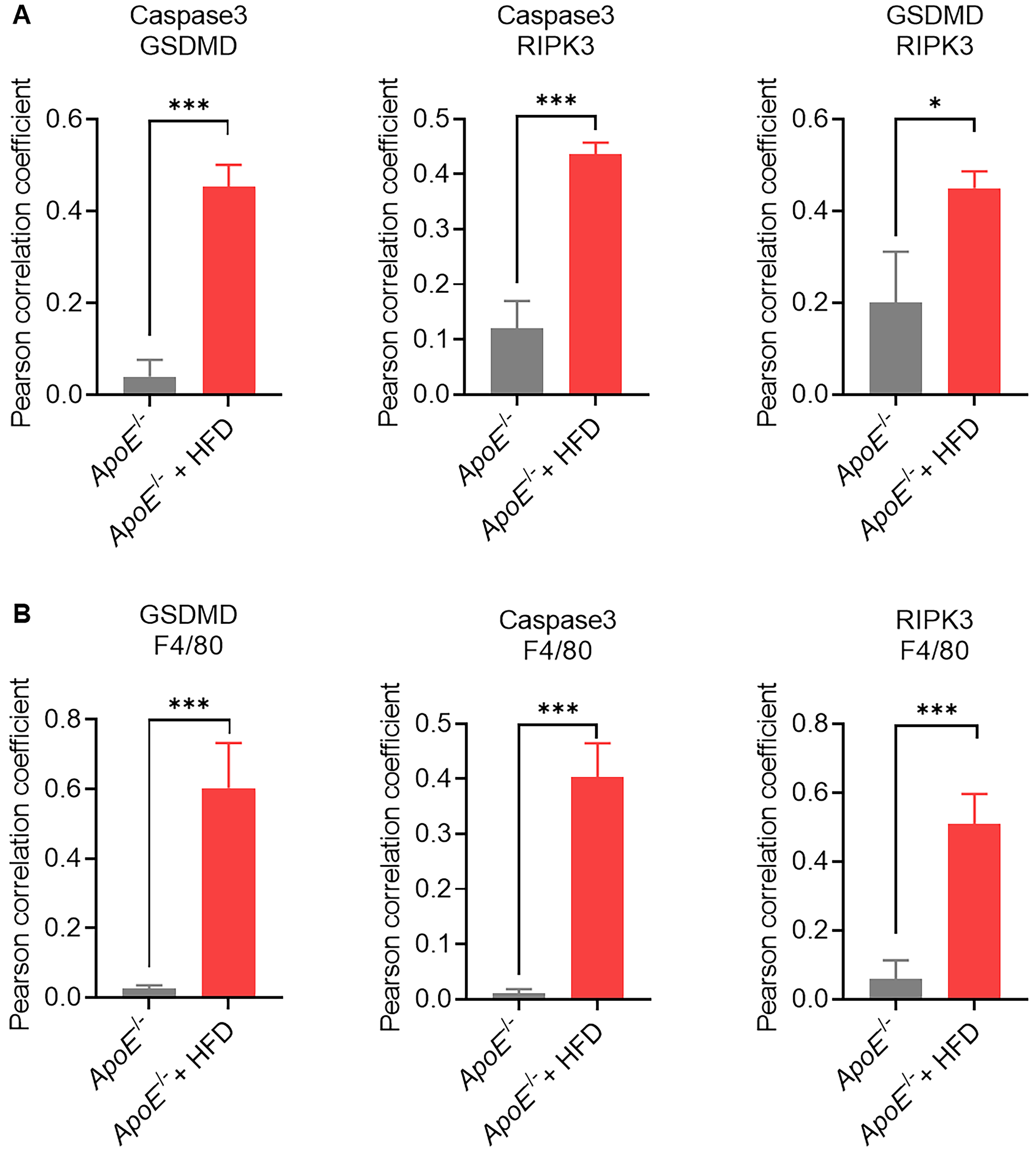

Supplement: Supplementary file 7 — Figure S7 Pearson correlation coefficients (PCCs) revealed the colocalisation of caspase‐3, GSDMD and RIPK3 in CD68‐positive macrophages within murine atherosclerotic lesions. (A) The colocalisation of caspase‐3/GSDMD, caspase‐3/RIPK3 and GSDMD/RIPK3 was evident in the atherosclerotic aorta of HFD‐fed ApoE−/− mice relative to the normal aorta of ApoE−/− mice. (B) The colocalisation of caspase‐3 with F4/80, GSDMD with F4/80 and RIPK3 with F4/80 were significantly enhanced in the atherosclerotic aorta of HFD‐fed ApoE−/− mice compared with the normal aorta of ApoE−/− mice. Data are derived from three to five independent experiments. * p ˂.05, ** p ˂.01, ***p ˂.001 by Student's t test. ns: not significant. [file CTM2-16-e70637-s003.png]

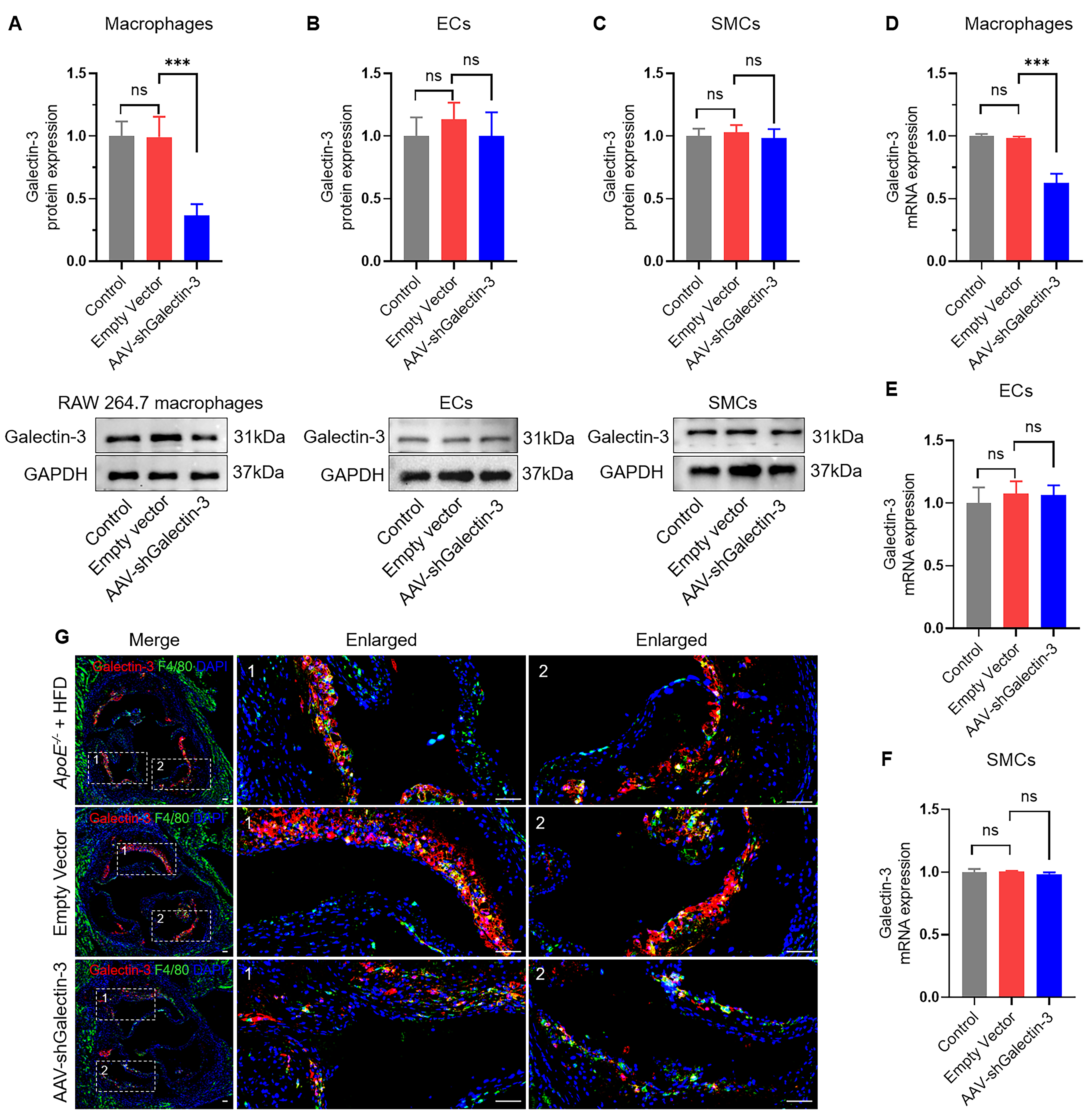

Supplement: Supplementary file 8 — Figure S8 AAV‐F4/80‐shGalectin‐3 specifically knocked down the corresponding protein in macrophages, while not affect SMCs and ECs in vivo. (A–C) In RAW 264.7 macrophages treated by AAV‐F4/80‐shGalectin‐3, we found a reduction in the protein levels of galectin‐3. In SMCs and ECs, after AAV‐F4/80‐shGalectin‐3, the protein expression of galectin‐3 is unchanged. (D–F) AAV‐F4/80‐shGalectin‐3 effectively and specifically downregulates the mRNA level of galectin‐3 specifically in macrophages, without altering its mRNA levels in ECs or SMCs. (G) Double immunofluorescence staining for galectin‐3 (red), F4/80 (green) and DAPI (blue) in the aortic root of HFD‐fed ApoE−/− mice demonstrates the location and distribution of galectin‐3‐positive macrophages as indicated by the colocalisation of galectin‐3 and F4/80 (a macrophage marker). Data are derived from three to five independent experiments. * p ˂.05, ** p ˂.01, ***p ˂.001 by Student's t test. ns: not significant. [file CTM2-16-e70637-s001.png]
